# Statistical Analysis of Molecular Signal Recording

**DOI:** 10.1371/journal.pcbi.1003145

**Published:** 2013-07-18

**Authors:** Joshua I. Glaser, Bradley M. Zamft, Adam H. Marblestone, Jeffrey R. Moffitt, Keith Tyo, Edward S. Boyden, George Church, Konrad P. Kording

**Affiliations:** 1 Department of Physical Medicine and Rehabilitation, Northwestern University and Rehabilitation Institute of Chicago, Chicago, Illinois, United States of America; 2 Department of Genetics, Harvard Medical School, Boston, Massachusetts, United States of America; 3 Biophysics Program, Harvard University, Boston, Massachusetts, United States of America; 4 Wyss Institute, Harvard University, Boston, Massachusetts, United States of America; 5 Department of Chemistry and Chemical Biology, Harvard University, Cambridge, Massachusetts, United States of America; 6 Department of Chemical and Biological Engineering, Northwestern University, Evanston, Illinois, United States of America; 7 Media Lab, Massachusetts Institute of Technology, Cambridge, Massachusetts, United States of America; 8 Department of Biological Engineering, Massachusetts Institute of Technology, Cambridge, Massachusetts, United States of America; 9 McGovern Institute, Massachusetts Institute of Technology, Cambridge, Massachusetts, United States of America; 10 Department of Physiology, Northwestern University, Chicago, Illinois, United States of America; 11 Department of Applied Mathematics, Northwestern University, Chicago, Illinois, United States of America; Accelrys, United States of America

## Abstract

A molecular device that records time-varying signals would enable new approaches in neuroscience. We have recently proposed such a device, termed a “molecular ticker tape”, in which an engineered DNA polymerase (DNAP) writes time-varying signals into DNA in the form of nucleotide misincorporation patterns. Here, we define a theoretical framework quantifying the expected capabilities of molecular ticker tapes as a function of experimental parameters. We present a decoding algorithm for estimating time-dependent input signals, and DNAP kinetic parameters, directly from misincorporation rates as determined by sequencing. We explore the requirements for accurate signal decoding, particularly the constraints on (1) the polymerase biochemical parameters, and (2) the amplitude, temporal resolution, and duration of the time-varying input signals. Our results suggest that molecular recording devices with kinetic properties similar to natural polymerases could be used to perform experiments in which neural activity is compared across several experimental conditions, and that devices engineered by combining favorable biochemical properties from multiple known polymerases could potentially measure faster phenomena such as slow synchronization of neuronal oscillations. Sophisticated engineering of DNAPs is likely required to achieve molecular recording of neuronal activity with single-spike temporal resolution over experimentally relevant timescales.

## Author Summary

Recording of physiological signals from inaccessible microenvironments is often hampered by the macroscopic sizes of current recording devices. A signal-recording device constructed on a molecular scale could advance biology by enabling the simultaneous recording from millions or billions of cells. We recently proposed a molecular device for recording time-varying ion concentration signals: DNA polymerases (DNAPs) copy known template DNA strands with an error rate dependent on the local ion concentration. The resulting DNA polymers could then be sequenced, and with the help of statistical techniques, used to estimate the time-varying ion concentration signal experienced by the polymerase. We develop a statistical framework to treat this inverse problem and describe a technique to decode the ion concentration signals from DNA sequencing data. We also provide a novel method for estimating properties of DNAP dynamics, such as polymerization rate and pause frequency, directly from sequencing data. We use this framework to explore potential application scenarios for molecular recording devices, achievable via molecular engineering within the biochemical parameter ranges of known polymerases. We find that accurate recording of neural firing rate responses across several experimental conditions would likely be feasible using molecular recording devices with kinetic properties similar to those of known polymerases.

## Introduction

When the monomers added to a growing polymer chain depend on signals in the environment, such as the ion fluxes during an action potential, the polymer sequence stores a record of the environmental signal's variation over time, much like a ticker tape [1], [2]. DNA polymerases (DNAPs), enzymes that catalyze replication of DNA, possess nucleotide misincorporation probabilities that can be modulated by local ion concentrations [3], [4], making them candidates for ion-sensitive molecular ticker tapes that encode signals into DNA strands in the form of base misincorporation patterns. For example, neural firing could be recorded by linking intracellular calcium concentration to polymerase misincorporation rates. In DNAP misincorporation-based recording, information is stored in the form of a string of copied nucleotides, which can be sequenced and compared to the known template sequence to identify the sites of misincorporations. Consequently, one can estimate the state of the environment – e.g. ion concentration – as a function of time, based on the observed misincorporation pattern.

A key problem for such biochemical ticker tape machines is that they may not have a high-fidelity clock. DNAPs do not add nucleotides at a constant rate [5], [6]: binding, catalysis, pausing, and dissociation from the template strand are thermally-activated, stochastic processes [7]. It is therefore necessary to address imperfect measurements of time in molecular ticker tapes.

**Figure 1:**
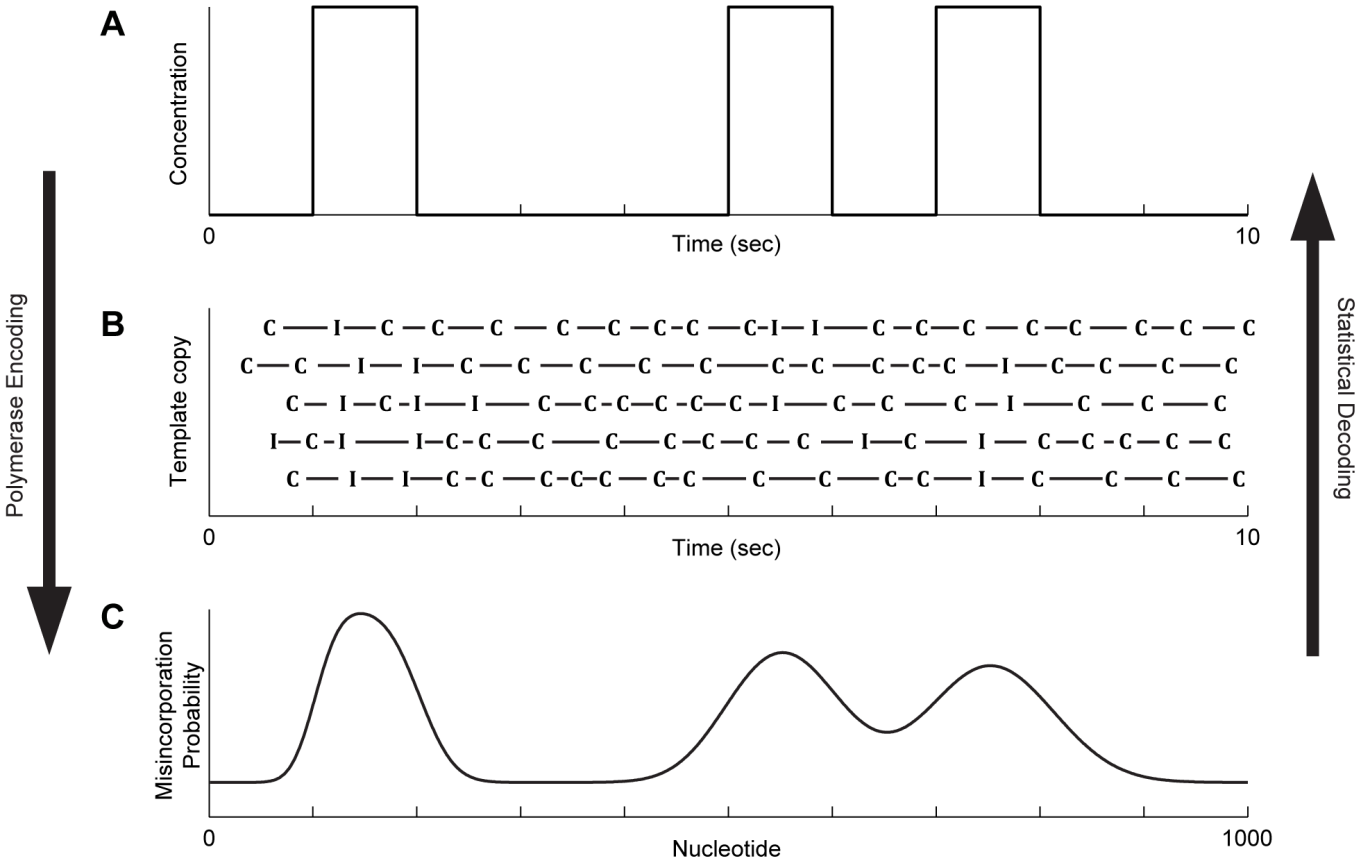
Encoding and decoding of signals with a molecular ticker tape. **A**) Example time-varying ion concentration signal. In a neuron, peaks in calcium concentration occur during neural firing. **B**) Example products from the simultaneous replication of multiple template strands, showing correct (C) and incorrect (I) nucleotide additions, with the time of incorporation shown on the horizontal axis. Misincorporations are more likely in the presence of higher ion concentration. **C**) The misincorporation counts from each template copy are summed to calculate the misincorporation probability at every nucleotide position in the template. In this example, approximately 100 nucleotides are replicated per second on average.

To assess the feasibility of extracting information from molecular ticker tapes, we analyze a system in which multiple ion-sensitive DNAPs simultaneously replicate identical DNA template strands in the presence of a time-varying ion concentration signal (Fig. 1A). In this scenario, DNAPs add each successive copied nucleotide with an ion concentration-dependent misincorporation probability. Due to thermal fluctuations, the time at which the addition of a particular nucleotide occurs must be treated as a random variable (Fig. 1B). In the limit of a large ensemble of simultaneously replicated templates, a misincorporation probability distribution can be measured as a function of the index of the nucleotide (Fig. 1C). Here we study the problem of estimating the ion concentration signal as a function of time, based on observed misincorporation frequencies as a function of the nucleotide index.

Our method for solving this inverse problem relies only on counting the total number of misincorporations as a function of position within the template. Therefore, it is directly compatible with current-generation short-read deep sequencing technologies, in conjunction with *in silico* sequence alignment algorithms (e.g. Smith-Waterman [8]), which would be used to localize the short reads inside a long, high-complexity DNA template sequence. Note that assembly of the short reads into contiguous strands, representing the output of a single polymerase molecule, is not required. This is fortunate because distinct error-prone copies of templates with identical sequences will share a high degree of homology and therefore may be difficult to assemble.

What are the biochemical properties that a DNAP must possess in order to function as a molecular ticker tape recorder? To allow for faithful decoding of realistic input signals, a DNAP may require a favorable combination of parameters such as speed, pause probability, distribution of pause durations, and ion-dependent misincorporation rate. Likewise, it is unclear how many simultaneously replicated template strands are required for accurate decoding.

Here we address these statistical constraints on molecular ticker tapes by presenting (1) an intuitive theoretical framework, based on Fisher information theory, which quantifies the theoretical optimal precision for estimating the time-varying input signal from sequencing data as a function of relevant biochemical and experimental parameters, and (2) decoding algorithms to perform estimation of the time-varying input signal from sequencing data. The decoding algorithms rely on knowledge of the DNAP's kinetic parameters. When these parameters are unknown, we provide an algorithm to calibrate them from sequence data generated in the presence of known input signals. Simulations of the decoding algorithm are used to determine the effects of relevant experimental parameters on the actual decoding performance of the algorithms (as opposed to their effects on the theoretical optima). With a view towards potential neuroscience applications, we identify polymerase parameter sets and input signal characteristics for which molecular recording may be feasible, thereby providing guidelines for the experimental design and validation of molecular recording technologies.

## Results

### Overview

The statistical feasibility of molecular recording depends on several experimental and biochemical parameters. We focus on (1) the kinetic parameters of polymerization by DNAP: the average single-base elongation time (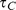), average pause time (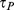), and pause probability (*P*); (2) the number of simultaneously replicated DNA template strands; and (3) the concentration to misincorporation link function (CMLF), which relates the per-base misincorporation probability to the local ion concentration. All these parameters can be determined experimentally prior to their use in molecular ticker tapes, either by traditional biochemical or single-molecule methods, or by those discussed below.

Using these parameters, we created a multi-parameter forward model (*Eqs. 2* *–* *5*; see *Methods*) for the probability of nucleotide misincorporation at any template base position, given a time-varying ion concentration signal. Based on this forward model, we derived an expression that analytically relates the optimal precision of ion concentration estimation to the model parameters in the setting of a single ion concentration pulse (*Eqs. 1* *&* *7*; see *Methods*).

For the case of realistic time-dependent ion concentrations, rather than single pulses, we have developed two algorithms (see *Methods*) to decode the time-varying ion concentration signal from the observed DNA sequences. The first algorithm estimates a continuous concentration trace by minimizing a cost function, while the second estimates a binary concentration trace using maximum likelihood estimation. A third algorithm determines unknown DNAP kinetic parameters from sequencing data, given known time-dependent ion concentration signals as inputs.

We first apply Fisher information theory to quantify optimal estimation precision for a single-pulse input, which results in a concise formula that provides intuition for the dependence of decoding fidelity on relevant experimental parameters. We next apply our decoding algorithms to simulated data. This allows us to quantify the achievable temporal resolution and recording duration of molecular ticker tapes in the context of realistic neural recording experiments. For several experimental paradigms, we determine the necessary DNAP kinetic parameters, CMLFs, and number of DNA templates. We also study the effects of DNAP dissociation from the template and of variation in polymerase start-times.

### Analytically relating estimation precision to experimental parameters

To provide some insight into the feasibility of ticker tape decoding under different experimental parameters, and to provide an analytical tool for testing the performance of our algorithms, we start by deriving the Fisher information associated with estimating the characteristics of a single concentration pulse from the observed misincorporation rate (see *Methods*). Here, the Fisher information 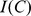 measures the degree to which the observed nucleotides are informative about the peak ion concentration *C* of an input pulse. A greater value for 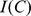 implies that 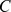 can be estimated more precisely: 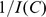 is the theoretical minimum variance of an unbiased estimator of 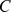 [9].

In the limit of small misincorporation rates, the Fisher information can be approximated as:
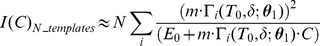
(1)(see *Methods*), where *N* is the number of DNA templates; 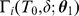 is the probability that nucleotide *i* was added during a concentration spike with start-time 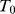 and duration 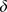, and DNAP parameters 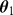; *C* is the ion concentration; 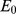 is the baseline error rate per base; and *m* is the slope of the CMLF, where we approximate the CMLF as linear [4], i.e., as 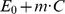.

*Eq. 1* confirms several natural intuitions about molecular recording: the theoretical optimal precision of ion concentration estimation can be increased by increasing *N* (the number of DNA templates; Fig. S1A), decreasing 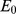 (the baseline misincorporation rate; Fig. S1B), increasing *m* (sensitivity of misincorporation rate to ion concentration changes; Fig. S1C), and increasing 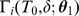 (probability that the 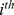 nucleotide was incorporated during the concentration spike). 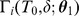 can be increased in multiple ways. Decreasing the pause duration or frequency increases 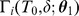 because polymerases will be less widely dispersed during the pulse when their nucleotide addition kinetics are less stochastic (Fig. S1D). Decreasing 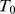 increases 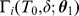 because the ensemble of polymerases de-phases over time (explained in more detail in *Methods*). Lastly, increasing 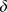, the duration of the concentration pulse, increases 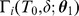. Note that, while *Eq. 1* applies in the limit of small error rates, the full expression for the Fisher information (*Eq. 7*) indicates that these general trends are still valid when considering moderate or large error rates; we use the full expression for the Fisher information in our simulations.

For further simplifications of *Eq. 1* in the limits of low and high baseline misincorporation rates and concentrations, see *Text S1: Further Simplifications*. We also studied how Fisher information governs the estimation of other properties of the concentration pulse in addition to its peak concentration: see *Text S1: Additional Pulse Properties*.

In the case of multiple concentration pulses, a Fisher information matrix can be constructed; however, this does not give rise to a simple analytic expression. Thus, to determine the performance of decoding multi-pulse input concentration traces, we implemented our decoding algorithms on simulated data in what follows.

### Testing the performance of decoding algorithms

Our continuous decoding algorithm, which minimizes prediction error by using a cost function, obtains ion concentration estimation variances similar to the Fisher information optimum when decoding a single concentration pulse (Fig. S1). When decoding more complex multi-pulse concentrations traces, the performance of this algorithm should be viewed as a lower bound on what could be achievable. Our binary decoding algorithm, which exhaustively computes the maximum likelihood concentration given the sequencing data, also obtains decoding accuracies similar to the Fisher information optimum when decoding a single concentration pulse, although its performance degrades relative to the theoretical optimum in the limits of small numbers of templates or high baseline misincorporation rates (Fig. S2). Theoretically, Fisher information naturally arises from maximum likelihood estimation [10]. Therefore, when determination of the maximum-likelihood concentration trace is possible, this simple decoding approach should be near optimal, even when decoding complex multi-pulse concentration pulses. Below we will use both ion concentration estimation algorithms to test the parameter requirements of molecular recording devices for neuroscience applications.

### Continuous concentration decoding

Many neuroscience experiments focus on measuring the firing rates of neurons. Understanding the factors that influence firing rates can inform researchers about what a neuron encodes. In order to test the ability of molecular ticker tapes to accurately record neural firing rates, we performed simulations using our continuous decoder, as increased firing rates will increase calcium ion concentration levels in a continuous manner [11] (further details about the conversion from calcium concentrations to firing rates can be found in the *Discussion*). We aimed to determine which biochemical parameters of a molecular ticker tape system are required to allow molecular recording of firing rates at the temporal resolutions characteristic of typical neuroscience experiments.

#### Recording firing rates across several conditions

Perhaps the simplest neuroscience experiments compare neural firing rates across several externally imposed conditions; for instance, to determine how neural firing rates differ in the presence vs. absence of a drug. There is a large class of such “multi-condition experiments”: examples include determining neural activity in response to varying behaviors, varying sensory stimuli (tuning curves), or systematic pharmacological, electrical, or optogenetic perturbations.

To test the feasibility of accurate molecular recording of a generalized multi-condition experiment, we considered a scenario in which multiple externally imposed conditions are presented in series over a period of time, while a molecular ticker tape records the time-varying ion concentrations resulting from the firing rates generated in response to each condition. We set the number of externally imposed conditions to eight, and the total experimental duration to 20 minutes, so that each condition lasts 150 seconds. Thus, in this scenario, a generalized multi-condition experiment corresponds to recording continuous ion concentration levels with a temporal resolution of 150 seconds for a duration of 20 minutes.

We used approximate DNAP kinetic parameters from 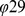 DNAP (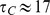 ms, 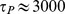 ms, 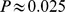) [12]. Note that these biochemical parameters change across experimental preparations, and the *in vivo* parameters in neurons are unknown, so this parameter choice may not always be accurate for 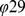 DNAP. We used a CMLF of 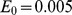 and 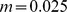, similar to that measured for Dpo4 in buffers of varying manganese concentrations [4], one of the few CMLFs experimentally measured at present. Note that while *m* generally has units of inverse concentration (e.g. M^−1^ or mM^−1^), here the concentrations in all simulations are scaled to range from 0 to 1 (arbitrary units), so that *m* also contains arbitrary units, and the misincorporation rate at high concentration is 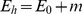 (here 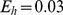). In our simulations, *m* can be viewed as the differential misincorporation rate, i.e., the difference between the misincorporation rates at high and low concentrations.

**Figure 2:**
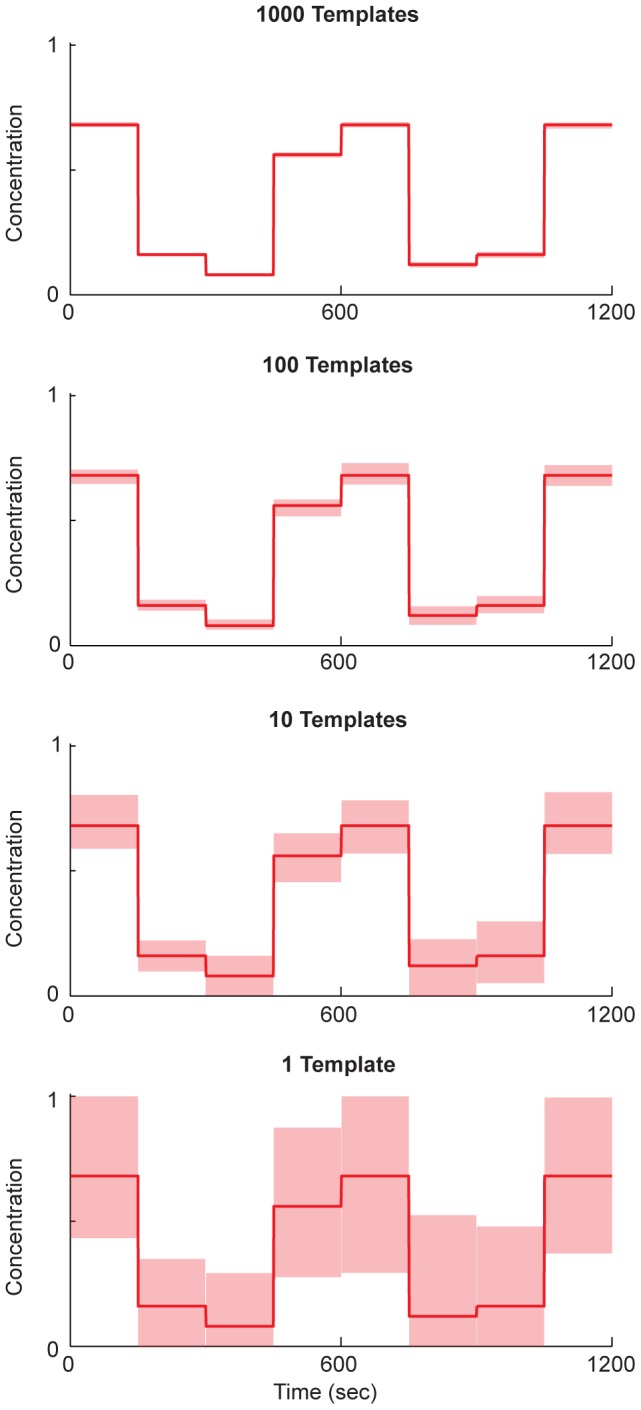
Decoding continuous concentration signals. Continuous decoding to estimate sequences of eight concentrations over 20 minutes of recording using varying numbers of templates. The 95% confidence interval of the estimated concentrations (light red) that result from the decoding algorithm presented here on an ion concentration input sequence representing the word “RECORDER” (dark red). Concentrations are mapped to letters via A = 0/25, B = 1/25,,…,Z = 25/25, so that the concentration sequence representing the word RECORDER is 17/25, 4/25…). The numbers of templates used were, from top to bottom, 1000, 100, 10, and 1. For all panels, kinetic parameters are those of 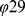 DNAP (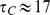 ms, 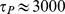 ms, 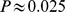), 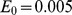, and 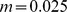 (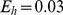).

We first tested the effect of varying the number of DNA templates on the accuracy of continuous concentration decoding at 150 second temporal resolution. An example is shown in Fig. 2, for a sequence of ion concentrations representing the word “RECORDER” (where the concentration of A = 0/25,…, Z = 25/25). In this example, with 1000 templates, concentration estimation is nearly perfect (1.8% median estimation error; Fig. 2).

**Figure 3:**
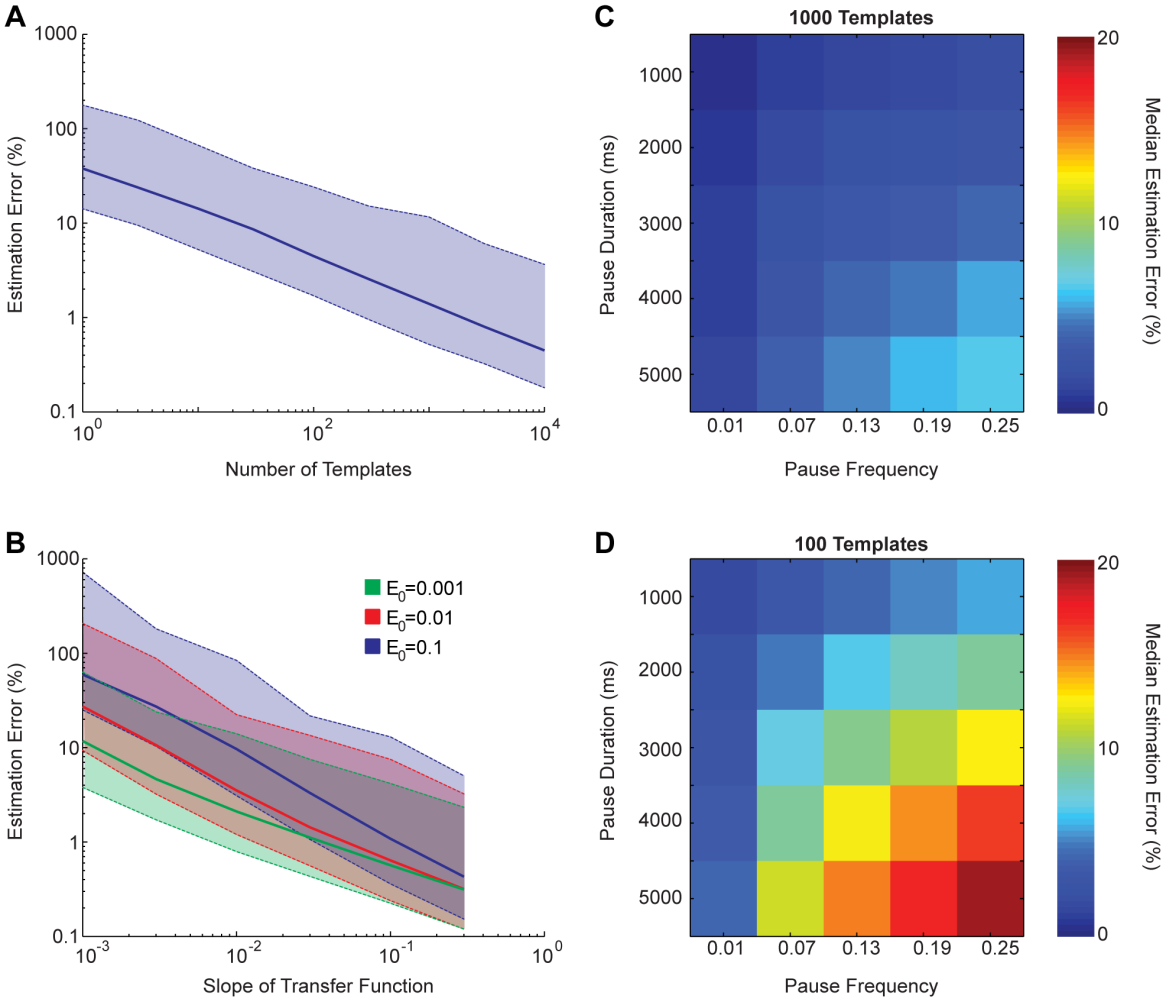
Varying numbers of templates, CMLFs, and DNAP parameters. Performance of continuous decoding to estimate randomly determined sequences of eight concentrations over 20 minutes of recording, as a function of experimental parameters. Solid lines are median estimation errors, and dashed lines are 95% confidence intervals. **A**) Varying numbers of templates, with the CMLF fixed at 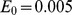 and 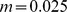, and using 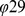 DNAP kinetic parameters. **B**) Varying CMLFs, with the number of templates fixed at 1000, and using 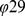 DNAP kinetic parameters. **C, D**) Varying DNAP pausing parameters, with a fixed elongation time of 20 ms, a fixed CMLF of 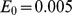 and 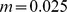, and 1000 and 100 templates, respectively.

Using randomly generated concentration sequences, we varied the number of templates (Fig. 3A), the CMLF (Fig. 3B), and the DNAP parameters (Figs. 3C&D). We found that multi-condition experiments could be performed using feasible numbers of templates and CMLFs, and DNAP parameters within the range of documented DNAPs. We also studied the effects of dissociation (Fig. S3), DNAP start-time variation, and concentration fluctuations (Fig. S4), and found these effects to be minimal in this context. Lastly, we studied the effect of varying the number of externally imposed conditions within the 20 minutes of recording (i.e., varying the temporal resolution), and found that approximately 10 conditions could be accurately recorded using 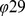 DNAP kinetic parameters, and more conditions with less stochastic parameters (Fig. S5). For a more in-depth explanation of our parameter sweep results, see *Text S2*. In general, we find that high accuracy molecular recording of multi-condition experiments is feasible using DNAPs with kinetic parameters similar to those of known polymerases.

#### Recording firing rates at 1000 ms and 100 ms temporal resolutions

**Figure 4:**
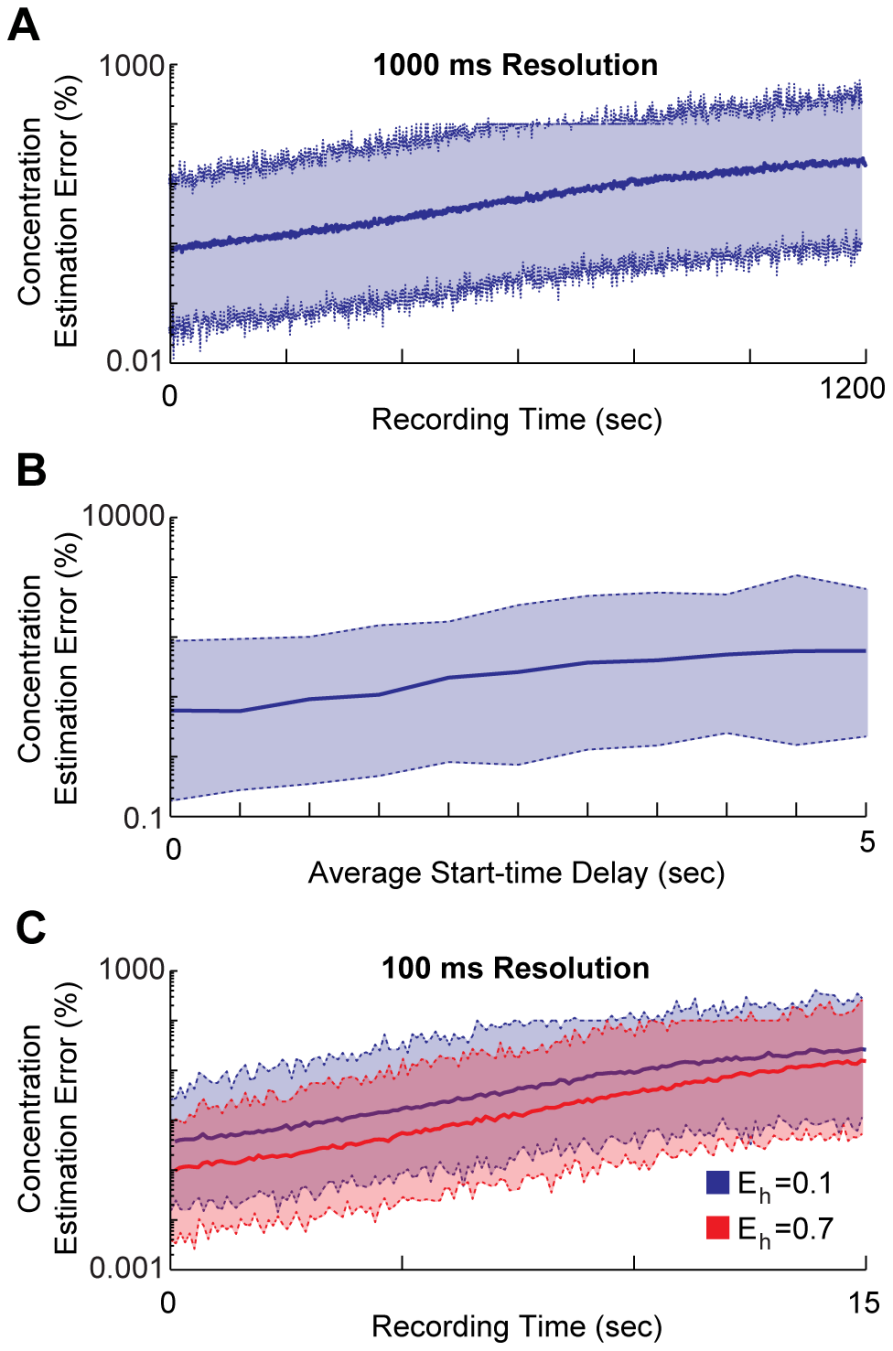
Continuous concentration decoding at high resolutions. **A**) Estimation error of continuous concentration decoding at 1 second resolution as a function of the time of recording. Parameters are 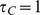 ms, *P* = 0, *N* = 1000, 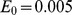, and 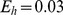. **B**) Estimation error at 6000 seconds (10 minutes) of recording for polymerases that do not start recording simultaneously. Polymerase start-time distributions are drawn from gamma distributions that have almost all values between 0 and twice the average delay time. **C**) Estimation error of continuous concentration decoding at 100 ms resolution as a function of the time of recording. Parameters are 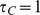 ms, *P* = 0, *N* = 10000, 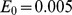, and varying 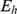. In all panels, solid lines are median estimation errors, and dashed lines are 95% confidence intervals.

Going beyond such generalized multi-condition experiments, which occur on a timescale of minutes, it is often of interest to study the dynamics of the firing rate at higher temporal resolutions, since many neuronal computations occur on timescales of 1000 ms (e.g. [13]) or less. What temporal resolutions are possible for continuous decoding using feasible biochemical parameters? Even with many templates (*N* = 10000), and the maximal differential misincorporation rate of 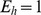 vs. 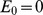, recording with 1 second temporal resolution yields over 50% median estimation error after only 5 seconds of recording when using 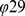 DNAP kinetic parameters. However, using optimal polymerase parameters consisting of a 1 ms elongation time (c.f., *E. coli* pol III [14]) and no significant pausing (e.g., T7 RNA polymerase [15], [16]), 1 second temporal resolution is possible for 10 minutes (6000 seconds) with ∼5% median estimation error (*N* = 1000, 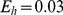, 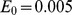; Fig. 4A). We further tested whether variation in polymerase start-times affected these conclusions. When polymerase start-times were allowed to vary from 0–2 seconds, median estimation error remained at <6% at 10 minutes of recording, but when start-times varied from 0–10 seconds, estimation rose to nearly 60% (Fig. 4B). As start-time variation can be large (e.g. shown to vary between 0.3 and 10 seconds *in vivo* in *Xenopus laevis* [17]), techniques such as optogenetics, which control molecular activities with <1 second temporal precision, will likely be required to decrease start-time variation. Thus, a DNAP constructed using a combination of the best parameters from within the range of documented DNAPs could likely be used to record continuous concentration traces at 1 second resolution, as long as polymerases are initially roughly synchronized.

Could such a DNAP record continuous concentrations at 100 ms resolution? Using a DNAP with a 1 ms elongation time and no pausing, 10000 templates, and a high differential misincorporation rate of 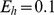 vs. 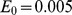, continuous concentrations can be accurately recorded (<5% median error) at 100 ms resolution for only about 8 seconds (Fig. 4C). Using 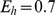 (polymerase Iota's misincorporation rate on template T [18]), accurate 100 ms resolution recording is still only possible for about 11 seconds. Non-synchronized start-times also have an even more deleterious effect at this higher temporal resolution: for example, when polymerase start-times vary from 0–1 seconds, the median estimation error is never below 30% (using 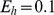). Start-time variation must be very small to have limited effect on recording accuracy: for instance, start-times that vary from 0–200 ms will allow <5% error until 7 seconds as opposed to 8 seconds. To record continuous concentration traces at 100 ms resolution for experimentally significant durations, sophisticated DNA engineering, to both lengthen the feasible recording duration and ensure extremely coordinated polymerase start-times, will likely be necessary.

### Binary concentration decoding

Some experiments seek only to determine whether or not a neuron has fired within a given time window, rather than to determine an analog firing rate. This binary, rather than continuous, decoding scenario could lead to different constraints on the biochemical parameters of molecular recording devices. We studied binary decoding in the context of two experimental paradigms: detecting synchronized firing and recording spike trains at single-spike temporal resolution.

#### Slow neuronal synchronization

Oscillations during slow-wave sleep are associated with frequencies of 0.1 to 0.5 Hz [19], while delta brain waves are associated with frequencies of 0.5 to 4 Hz [20]. A binary decoder with 100 ms temporal resolution could map such synchronization by determining whether any pair of neurons consistently fired together during 100 ms intervals.

We investigated the CMLFs required for this application, using optimal DNAP kinetic parameters from within naturally known ranges (1000 nt/sec elongation rate, no pausing, no dissociation) and 10000 DNA templates. For 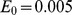 and 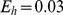, binary decoding at 100 ms temporal resolution could be achieved for a recoding duration of 325 seconds at 95% accuracy (Table 1). A 10% misincorporation rate at high ion concentration could provide the same level of resolution and accuracy for over 700 seconds of recording (Table 1). We again find that for a constant ratio of misincorporation rates at high and low ion concentrations (diagonal of Table 1), increasing misincorporation rates increases the feasible duration of recording. Additionally, decreasing the speed of the polymerase has a strong effect: an elongation time of 10 ms (as opposed to 1 ms), decreases the feasible recording time from 300 seconds to 10 seconds (at 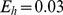).

**Table 1:** Binary decoding at 100 ms resolution.

	Baseline Misincorporation Probability (*E*_0_)			
Misincorporation Probability at High Concentration (*E*_h_ = *E*_0_+*m*)	0.5%	1.5%	5%	15%
1%	75 sec			
3%	325 sec	125 sec		
10%	700 sec	475 sec	250 sec	
30%	1275 sec	1000 sec	750 sec	425 sec

The maximum recording duration at which decoding at 100 ms temporal resolution is possible with 95% decoding accuracy. An optimal DNAP with an elongation time of 1 ms and no pausing is used, along with 10000 DNA templates. The search for maximal achievable recording durations was performed at 25 second intervals.

We next tested the effect of varying start-times on 100 ms resolution binary decoding. As was the case for continuous decoding, we found that start-time variation has a large impact on the feasible recording duration at this resolution. Start-times varying between 0 and 1 seconds still allow 95% decoding accuracy until 300 seconds of recording (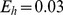). However, for start-times varying between 0 and 3 seconds, 95% decoding accuracy is never achievable. Techniques that decrease start-time variation would thus be necessary in order to use molecular ticker tapes to record slow synchronization of neuronal oscillations.

Although these experiments would be limited to hundreds of seconds, the large number of individual neurons that could potentially be recorded could provide fundamentally new insights into mechanisms of neural synchronization. We thus find that coarse measurement of neuronal oscillations could be feasible at the limits of documented polymerase parameters, assuming an ample number of simultaneously replicated templates per cell and a mechanism to control the polymerase start-times.

#### Single-spike resolution

A desirable application for molecular ticker tapes would be the recording of neuronal spike trains at single-spike resolution (approximately 10 ms), e.g. for the study of spike timing dependent neural coding and plasticity [21]. A binary decoder would be sufficient to determine whether or not a neuron has spiked within a 10 ms time bin.

Would a DNAP constructed from optimal kinetic parameters found within natural polymerases (1000 nt/s speed, no pausing) be able to record at 10 ms resolution? We find that only one second of recording with 95% accuracy is possible when 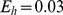, 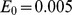, and there are 10000 templates. If the misincorporation rate at high ion concentration is increased to 10%, then ∼2.5 seconds of recording at 10 ms temporal resolution could be achieved. In order to achieve 1 minute of accurate recording, a polymerase with a speed of 8000 nt/s (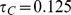 ms) would be required given a 10% high misincorporation rate. Even in the limiting case of a 100% high misincorporation rate and a 0% low misincorporation rate, with no pausing and 10000 templates, a speed of 3500 nt/s would still be needed to achieve 1 minute of recording at 10 ms temporal resolution. These speeds are outside the range of polymerase speeds known from nature.

Therefore, even in the absence of pausing, and with arbitrarily high signal-to-noise ratio in the ion-dependent misincorporation rate, temporal stochasticity constrains the achievable temporal resolution for molecular recording. This results from the fact that there is no deterministic one-to-one mapping between time and nucleotide position; the time between base additions in the elongating state is not a constant but is rather governed by a probability distribution over dwell times. Our results suggest that recording spike trains at 10 ms resolution with a DNAP misincorporation-based molecular ticker tape and short-read sequencing, for more than a few seconds, would require sophisticated protein engineering to go beyond naturally occurring polymerase parameters.

### Calibrating unknown DNAP parameters via sequencing

**Figure 5:**
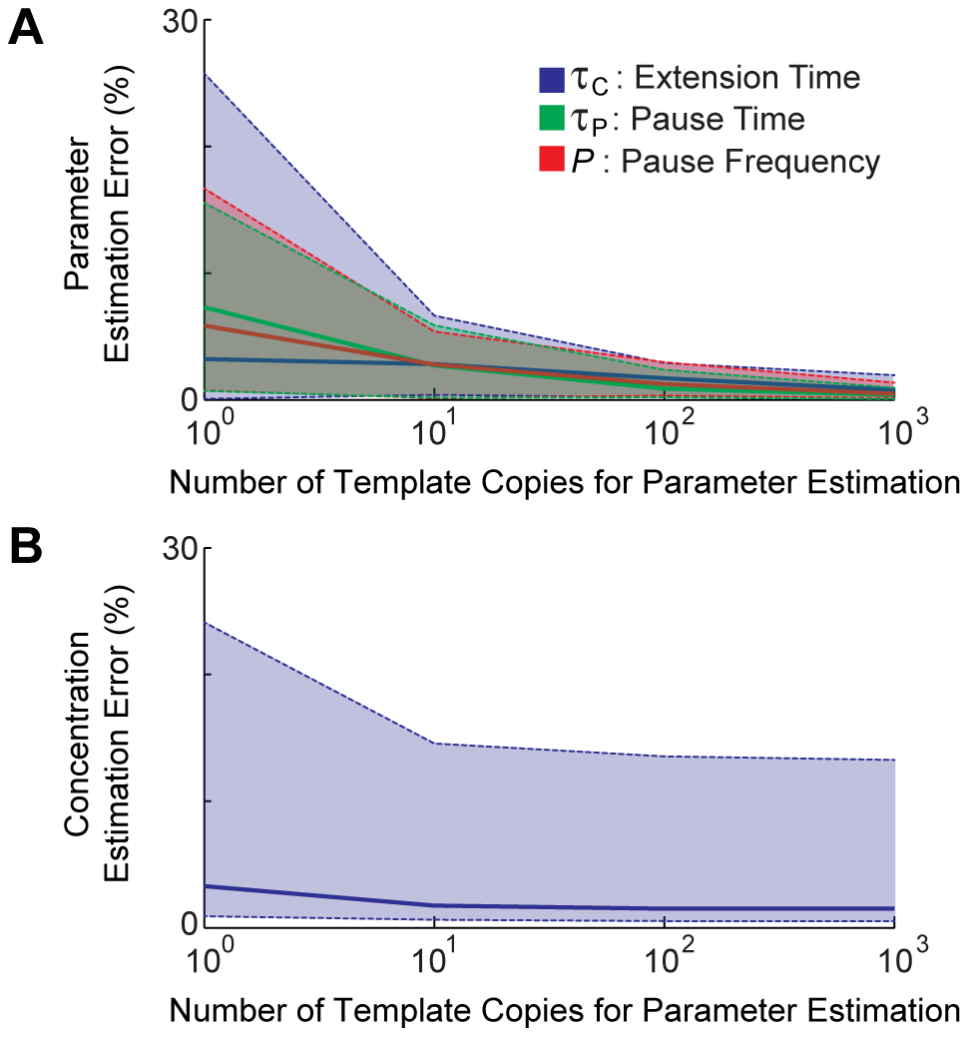
Estimating DNAP parameter values from sequencing data. **A**) The percent error of the estimated parameters compared to the true parameters (those of 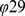 DNAP) as a function of the number of template copies. **B**) The ion concentration estimation error based on polymerase parameters estimated from data using varying numbers of templates. Ion concentration estimation used *N* = 1000, 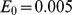 and 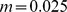. In both panels, solid lines are median estimation errors, and dashed lines are 95% confidence intervals.

Decoding unknown input signals requires a detailed model of the polymerase dynamics (see *Text S3*, Fig. S6); however, such information may not always be available *a priori*. To determine if it is possible to calibrate the polymerase parameters from sequencing data generated with a known input signal, we tested the accuracy of estimating the three kinetic parameters of 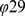 DNAP with varying numbers of template copies for a fixed input concentration sequence of 10010001, with each segment lasting 150 seconds (the timeframe we use when analyzing multi-condition experiments). The percent error of the estimated parameters relative to the true parameters decreased as the number of template copies increased, with an especially sharp drop from 10 to 100 templates (Fig. 5A). Thus, it should be possible to calibrate polymerases with high accuracy, *in vivo*, where their dynamic properties may not be known.

To confirm that polymerase parameters calibrated as above could be used to decode novel signals, we tested concentration decoding by using the estimated parameters on new (i.e. unknown) input signals. Specifically, we performed continuous ion concentration estimation in the multi-condition experiment framework as before, using 1000 DNA templates, and a CMLF of 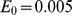 and 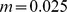, but this time using estimated 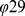 DNAP parameters as opposed to those used while producing the forward model. When we used at least 1000 templates to calibrate the DNAP parameters, we were able to estimate the initial time-varying ion concentration with ∼1.5% median error (Fig. 5B), a minimal change compared to the error obtained using known parameters. Our results indicate that data driven calibration is an effective method that will allow decoding of concentration traces generated using previously un-characterized polymerases (i.e., those that have not been subjected to single-molecule biophysics experiments to determine their detailed properties in physiologically relevant contexts).

## Discussion

This work presents analytical and algorithmic approaches to the statistical problems associated with signal reconstruction in molecular ticker tapes. We develop a procedure to estimate the time-dependent state of the environment from the observed strings of symbols in the replicated polymers and analyze its dependence on several experimentally relevant parameters. In addition, we present an analytical approach that illustrates intuitively how the precision of ion concentration estimation depends on these parameters. We show that high-accuracy estimation is possible under certain conditions even for DNAPs that dissociate from the DNA template and those with asynchronous start-times.

### Comparisons with existing neural recording technologies

A proposed application for molecular ticker tapes is to record simultaneously from large numbers of neurons. In the scheme treated here, polymerase misincorporation rates would be made to depend on ions, such as calcium, for which the intracellular concentration varies in response to neuronal activity. More generally, physical variables such as membrane voltage could be used in a molecularly engineered system to modulate nucleotide incorporation probabilities either through direct physical action on the polymerase, or indirectly by controlling the availability of small molecules such as nucleotides or ions. It should thus be possible to use DNAPs to measure many microscopic variables of interest in neuroscience.

It is important to compare the spatial resolution of molecular ticker tapes to existing techniques. One highly scalable technique with high spatial resolution is 2-deoxy-D-glucose (2-DG) imaging [22], which utilizes the fact that active neurons consume more glucose, and allows estimation of neural activity during one or two [23] conditions. Molecular ticker tapes promise to achieve similar spatial scale and resolution to 2-DG, while also allowing multiplexing across many conditions during the same experiment. In particular, our results suggest that molecular ticker tapes could be used to determine the firing rate responses of neurons under a sequence of ∼10 conditions, using a DNAP with kinetic parameters similar to those of 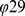 DNAP. A popular technique with lower spatial resolution (∼1 mm) is fMRI, which is often used to compare voxel-scale hemodynamic responses across a number of conditions. Molecular ticker tapes, in contrast, promise much greater spatial resolution while also allowing multiple conditions per experiment.

It is also important to compare the temporal resolution of molecular ticker tapes to existing techniques. We found that, while determining binary neural responses could be feasible at resolutions of >10 Hz through combining favorable biochemical parameters from multiple existing DNAPs into a single engineered system, temporal resolutions approaching 100 Hz may be hard to reach, for recording durations of longer than a few seconds, without sophisticated protein engineering to go beyond individual parameters known from nature. However, molecular ticker tapes do have the potential to rival the temporal resolution of fMRI and surpass that of 2-DG imaging. Many other techniques, including EEG, local field potentials, calcium imaging, and single cell recordings, allow very high temporal resolution but are currently limited to small numbers of simultaneously recorded cells; greatly improved engineered DNAPs would be necessary for molecular ticker tapes to reach comparable temporal resolutions. Molecular ticker tapes thus present an opportunity to combine effectively unlimited spatial resolution with temporal resolution sufficient for complex functional studies, but this approach will face challenges in capturing the single-spike timescale (see Table 2).

**Table 2:** Technology comparison.

	Spatial Resolution	Temporal Resolution
**fMRI** (current setups)	∼1 mm	∼1 sec
**2-DG**	∼100 µm	>30 min
**Ticker Tape** (DNAP kinetics at limits of known polymerases, 10k DNA templates)	1 neuron (∼10 µm)	∼100 ms binary decoding ∼1 sec continuous decoding

Approximate spatial and temporal resolutions for a subset of technologies theoretically capable of recording from entire mammalian brains.

### Estimating DNAP kinetic parameters using molecular recording

There is considerable uncertainty about the parameters characterizing the dynamics of most DNAPs. Databases available online [24] do not fully specify the dynamics. More importantly, there may be significant variation in polymerase dynamics between different *in vivo* settings. Our study suggests that DNAP kinetic parameters can be characterized using data solely derived from deep sequencing. We could fit average speed, processivity, misincorporation rates (insertion or deletion), pause density and duration, and any other such parameters. These parameters could even be determined in a high throughput manner from sequencing data as a function of many variables such as divalent cation concentration [4], substrate composition/concentration, or the effects of inhibitors/mutagens. The amenability of this sequencing-based characterization method to high-throughput experimental procedures stands in contrast to more traditional single-molecule methods. Additionally, this approach could allow determination of *in vivo* DNAP dynamics, which is usually not accessible to single-molecule methods. The method we present, based on fitting a generative model using sequence data, may therefore augment methods based on direct single-molecule biophysical observations of the dynamics of polymer-generating molecular machines [12], [25]–[28].

Here we have focused on a DNAP-based molecular ticker tape. However, our methodology could be applicable to any molecular recording device based on modulating a polymerization process. Any system in which an enzyme catalyzes the formation of polymers with sequence features dependent on an environmental signal will fit into our basic theoretical framework and could be used to record time-varying signals. Thus, our methodology could also be used to characterize kinetic parameters of enzymes besides DNAP.

### Algorithmic limitations and future directions

While neural spike times and firing rates are the variables critical to neuroscience, our continuous decoding algorithm estimates time-dependent concentrations. Sometimes, calcium concentration increases linearly with firing rates. For example, sustained firing in the proximal apical dendrite of cortical layer 5 pyramidal neurons results in a calcium concentration that scales approximately linearly with firing rate (its equilibrium time constant is 200 ms) [11]. However, the conversion from intracellular free Ca^2+^ concentrations to spike rates is generally nonlinear and dependent on cell type (e.g. on the number and distribution of voltage gated calcium channels). Nonetheless, the problem of inferring spike rates from Ca^2+^ signals has been studied extensively and effective algorithms have been developed [29]–[31]. Intracellular free Ca^2+^ has been successfully used as an indicator of neural activity in many high-resolution techniques [32], [33]. If ion-dependent DNAP misincorporation is used as a sensing mechanism in molecular ticker tapes, then future work will need to combine our methodology with these techniques to infer neural firing rates from ion concentration traces. However, it may also be possible to directly couple DNAP misincorporation to trans-membrane voltage through the use of protein engineering.

The conclusions from our binary decoder regarding what is feasible are based on the assumption that the maximum likelihood solution can be found. While we performed an exhaustive search of the binary parameter space to find the maximum likelihood solution, to use this algorithm for long bit strings, more efficient binary optimization routines will be necessary.

The algorithms used here make important assumptions about DNA replication. They assume that misincorporation probabilities at neighboring template bases are statistically independent, an assumption that significantly simplifies all calculations and that is consistent with previous measurements in the presence of fixed concentrations of manganese [4]. This assumption could be violated if the misincorporation rate at a base depends on the presence or absence of a mismatch across the double helix at the previous base. Such effects would need to be incorporated into the forward model of polymerase misincorporations as well as the decoding algorithms.

Another conflating effect could also occur under time-varying (but not static) ion concentrations, if the elongation time at a base depends on the presence or absence of a misincorporation (and hence a mismatch across the double helix) in the previous position(s). Further studies are needed to address the effect of nearby mismatches on the nucleotide addition time and the interaction of this effect with time-varying ion concentrations.

Furthermore, the algorithms used here assume that the DNAP dynamics (e.g., elongation rate, pause rate and pause duration) are unaffected by the surrounding ion concentrations (except via the misincorporation probability itself that is deliberately a function of ion concentration), which is in general not true [15], [18], [34]. In a plausible alternative scenario, the elongation time may depend directly on the instantaneous local ion concentration. While this would not directly couple misincorporations at adjacent nucleotides via the forward model (i.e., the DNAP's misincorporation probability at a given time still depends only on the instantaneous ion concentration, and not on its history of previous misincorporations), it would lead to changes in polymerase dynamics over time, causing increased variation in the incorporation times for the 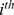 nucleotide. It would also lead to a more difficult inverse problem, as the misincorporation rate at a given nucleotide position would depend on the entire history of the unknown input ion concentration trace that is to be estimated. In future work, this feature could be added, motivating an EM-type algorithm [35] which iteratively adjusts both the forward model parameters (as a function of ion concentration) and the inferred ion concentration trace itself.

Our methods also assume a given temperature and fixed concentrations of DNA template, DNAP and nucleotide substrates, and do not account for local template structure or for the identity of the nucleotide to be copied, all of which are important [4], [26], [36]. However, these features could be readily accounted for by adding more parameters to the model. Despite these assumptions, the decoding algorithms are simple and applicable to the problems defined by current experimental techniques, e.g., in the context of preliminary experimental testing of molecular recording paradigms.

Real polymerase kinetics are more complex than our simple forward model, in which the sum of two exponentials governs the time distribution between nucleotide additions. In principle, any model of DNAP dynamics [12], [37] could be fit to the same data. It may also be possible to generalize this work based on general statistical descriptions of enzymatic dynamics [38]–[40]. This approximation approach would decrease our algorithm's run-time and the amount of data required. In the future, the methods presented here can thus be extended to treat more realistic enzyme kinetics.

Lastly, the inverse problem that we are solving here has deep connections with deconvolution, and one could argue that misincorporation rates result from a time-dependent convolution of a time-dependent source signal. There is a rich literature on such deconvolution techniques (e.g. [29], [30], [41], [42]) and more generally on latent variable models, which have been prominently used in neuroscience (e.g. [43]–[45]). Combining our approaches with existing computational methodologies promises to enable improved algorithms.

### Towards single-spike resolution

Could molecular signal recording at high temporal resolutions be possible? Increasing the polymerase speed and decreasing the stochastic pausing of an engineered DNAP might be the most feasible pathways. At a more fundamental level, one could engineer a DNAP to exhibit less stochasticity in its elongation rate (i.e., compared to the assumption of a single-exponential distribution of dwell times in the elongating state which was studied here) even after pausing has been eliminated. In general, multi-step kinetic processes can be remarkably regular in time, as long as the rates of each of the kinetic sub-steps (e.g., nucleotide entry, binding, pyrophosphate cleavage, pyrophosphate release, and physical motion of the enzyme forward) are comparable [7]. For example, the packaging motor of the bacteriophage 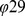 moves along DNA with little stochasticity under certain adenosine triphosphate concentrations because as many as six separate kinetic events are equally rate limiting in its catalytic cycle [46], [47]. Similarly, one could imagine engineering clock-like polymerases by first removing pause states and then by balancing the rates of the multiple catalytic sub-steps within each nucleotide addition. Thus, there are polymerase engineering directions that could significantly improve temporal resolution.

While we assumed here a single-exponential dwell time distribution in the elongating state, to our knowledge this distribution has not been experimentally measured. Thus, it is possible that real polymerases are less stochastic at the level of pause-free nucleotide additions than we have assumed. If true, this could make the problem of engineering a molecular recorder with single-spike resolution more tractable.

Combining the methodology discussed here with additional experimental and computational machinery may also significantly enhance the achievable temporal resolution. For instance, an external signal could be used as a clock pulse. Neurons could be optogenetically activated at known times, altering the misincorporation rates of nucleotides incorporated at those times, effectively embedding synchronization signals into the DNA sequences. Computational techniques could be developed to estimate the nucleotides' incorporation times given this additional timing information. As our methodology is currently limited by the stochasticity of nucleotide incorporation times, such an approach would have the potential to increase the feasible duration of recording at high temporal resolutions.

### Conclusion

The ability to record cellular signals is a cornerstone of neuroscience. Current macroscopic recording devices can simultaneously sample only a tiny number (currently hundreds to thousands) of neurons in mammals [48], [49] (but see [13]). Due to the scalability of molecular technology, the molecular signal-recording devices discussed here could potentially enable the simultaneous recording from millions or billions of neurons. This approach is particularly attractive because the price-performance of DNA sequencing has been improving faster than Moore's law [50]. Statistical techniques to allow precise readout, despite the imperfect clocks of molecular ticker tapes, will be important for the development of molecular recording technologies. In demonstrating these computational techniques, we have illustrated an analytical framework as well as practical decoding methods that provide insight into the capabilities and limitations of molecular ticker tapes as a function of relevant experimental parameters.

## Methods

### Derivation of the forward model: Modeling DNAP dynamics and dwell time distributions

**Figure 6:**
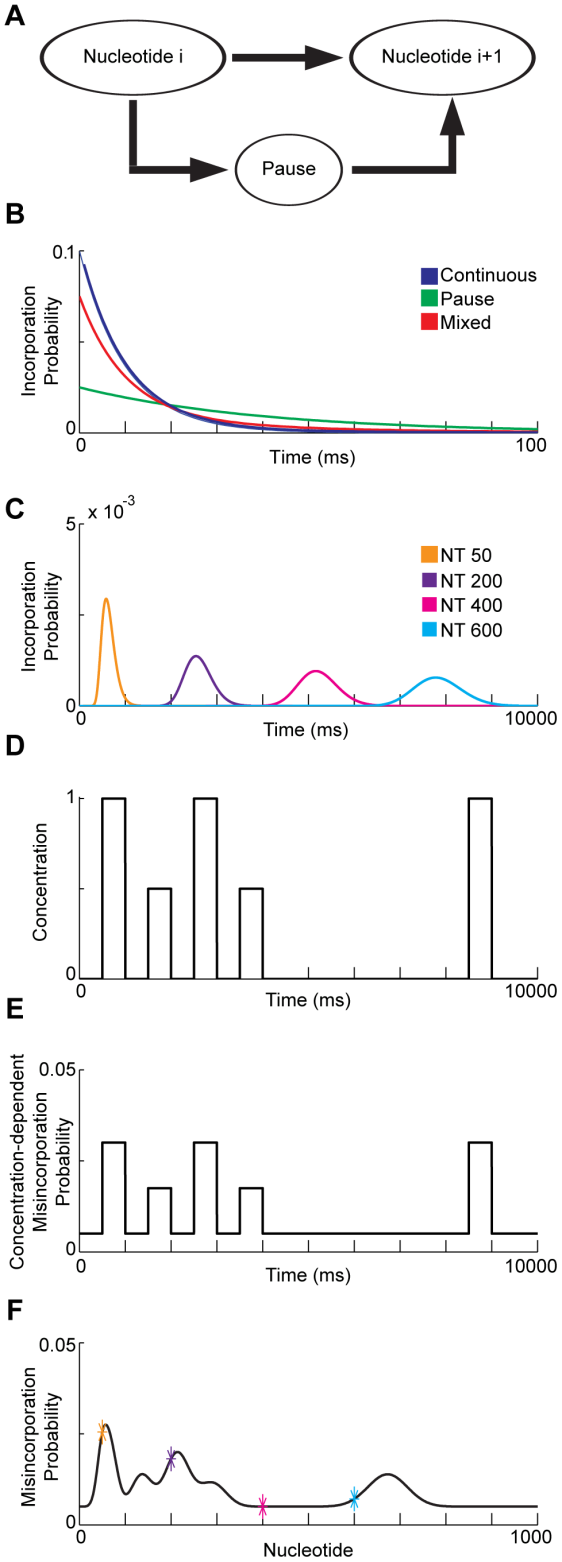
Minimal forward model of misincorporation by a DNAP. **A**) DNAP can copy one nucleotide directly after another (top path) or pause between additions (bottom path). **B**) Dwell-time distributions between nucleotide additions. Distributions for the continuous route and for the pausing route are mixed based on their relative frequencies to create the full dwell time distribution, 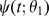. For this panel, the parameters are set as 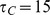 ms, 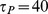 ms, and *P* = 0.3, to best illustrate the concept of distribution mixing. **C**) Time distributions, 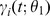, resulting from repeated convolutions of the dwell time distribution, are shown for nucleotides 50, 200, 400, and 600. Iterated convolutions cause the distribution to widen for later times. For this panel and below, parameters are 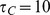 ms, 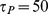 ms, 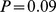. **D**) An example time-varying concentration. **E**) The probability of misincorporation for a polymerase subjected to the input concentration trace from panel B. The misincorporation probability is related to the concentration through a CMLF: here, 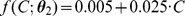 **F**) The misincorporation probability of the 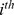 nucleotide, 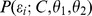. The more the 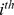 nucleotide's incorporation-time distribution overlaps with the concentration peaks in the time-varying input signal, the larger the misincorporation probability at the 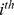 nucleotide.

We use a simplified model of DNAP dynamics based on recent single-molecule measurements [37]. We model the time distribution between successive nucleotide additions, or “dwell time,” as the sum of two exponentials (Fig. 6A&B), which correspond to the processes of (i) continuing directly from one nucleotide to the next, and (ii) pausing in an off-pathway state between nucleotide additions. A decaying exponential has been recently shown to fit pause lifetime data [6], [51]. The normalized probability distribution over times between successive nucleotide additions is then:
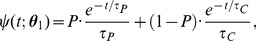
(2)where 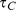 and 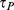 are the average times for the continuous (elongation) and pausing paths respectively, *P* is the DNAP pause probability per nucleotide (i.e. the pause density), and 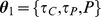 is the parameter set for a particular DNAP.

For a full discussion of polymerase model simplifications, see *Text S4*. Importantly, while we have here chosen to approximate dwell time as the sum of two exponentials, any normalized dwell time distribution is compatible with our methodology, as any two probability distributions can be convolved (see below).

### Derivation of the forward model: Time distributions of nucleotide incorporations

The probability distribution over dwell times that we discuss above induces a probability distribution for the time of the 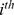 nucleotide addition, 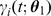. Basically, the time nucleotide 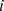 is written is the sum of the time nucleotide 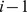 was written plus the dwell time drawn from the distribution 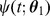 (*Eq. 2*). We calculate the probability distribution of the sum of two independent random variables (we assume in this model that the dwell time distributions for subsequent steps are independent) via the convolution of their distributions [52]:
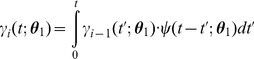
(3)(Fig. 6C). Note that unless otherwise stated, polymerases are assumed to start at the first nucleotide at time 0, so that 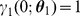.

For large *i*, 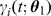 can be approximated as a Gaussian (as is done in the enzymatic dynamics literature [38]–[40]): 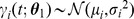. This can be expressed for every nucleotide *i*, in terms of the DNAP kinetic parameters:
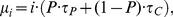
(4a)

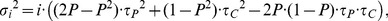
(4b)where 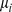 and 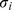 are respectively the mean and standard deviation of a Gaussian distribution governing the time at which nucleotide *i* is incorporated. For all simulations we will work with a discrete-time form of 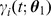, and use this Gaussian approximation for large *i* (see *Text S5* for details). The time 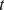 when each nucleotide is written is a latent variable here, which we integrate out (see *Eq. 5*).

### Derivation of the forward model: Quantifying misincorporation probabilities

A concentration to misincorporation link function (CMLF), *f*, quantifies the misincorporation rate when a nucleotide is replicated in the presence of a constant concentration. We assume this function to be known based on previous experiments. In molecular recording, a nucleotide will be copied as concentrations are fluctuating. Thus, in this simplest of models, a nucleotide's probability of misincorporation is dependent on the ion concentration at the time at which it is incorporated, and on the CMLF. To calculate the probability of misincorporation on the 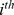 nucleotide, we weight the probability of misincorporation resulting from the ion concentration at a given time *t*, 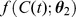, by the probability 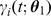 that the 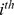 nucleotide is copied at time *t*, and sum this product over all values of the time variable (marginalization):
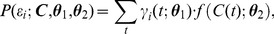
(5a)where 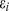 is a misincorporation on nucleotide *i*, 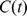 is the ion concentration at time *t*, and ***C*** (bolded) is the vector with elements 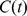 over all times *t*.

We assume *f* is linear (Fig. 6D&E) [4], i.e.
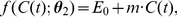
(5b)where 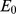 is the baseline error rate, *m* is the slope of the CMLF, and 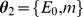. In this case,
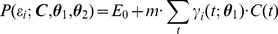
(5c)(Fig. 6F). Note that the linear form of the CMLF generally is approximately accurate for small concentration perturbations, as 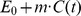 is the first-order Taylor expansion of a general, smooth CMLF.

### Analytical relation between estimation precision and experimental parameters

Fisher information measures the degree to which samples from a probability distribution are informative about the parameters characterizing that distribution. In the simplified case that there is a single ion concentration square pulse during a time interval starting at time 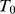 with duration 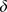 (the ion concentration assumed zero elsewhere), we analytically quantify the Fisher information, 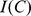, that *N* copied DNA templates contain about the concentration, 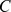, during time 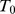 to 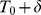.

Applying the previously derived forward model (*Eq. 5c*), we set the probability of misincorporation at the 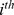 nucleotide as 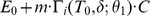, where 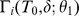 is the probability that nucleotide *i* is replicated during the time interval at which the concentration burst is present:
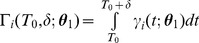
. From now on, we will refer to 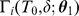 as 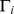 for brevity. We let 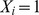 signify a correct incorporation at nucleotide *i* and 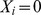 signify a misincorporation at nucleotide *i*, so that the probability of 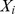 is
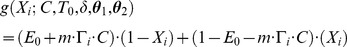
(6)From now on, 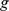 will be written only as a function of parameters that are explicitly changing (in this case 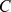).

The Fisher information in this distribution is:
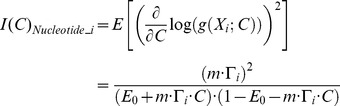
(7a)At constant ion concentrations, misincorporation probabilities at successive template bases are approximately independent [4]. Because Fisher information is additive across independent events,
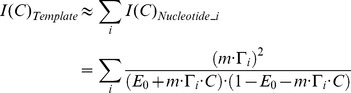
(7b)Additionally, we assume that individual templates are copied independently, so that:
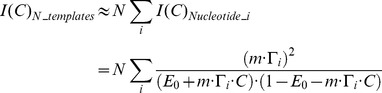
(7c)We use *Eq. 7c* as a basis for our analysis.

In the limit that 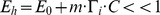, the Fisher information can be approximated as:
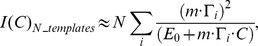
as is given in *Eq. 1*.

For a method to determine optimal decoding accuracy of a single concentration pulse using Fisher information, expressions of how Fisher information relates to pulse properties besides concentration, and full derivations, see *Text S1*.

### Estimation of continuous time-varying concentrations

In order to move beyond the assumption that there is a single concentration pulse, we estimate the concentration trace by minimizing a convex, differentiable, cost function with constraints:
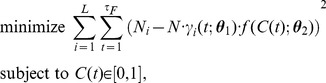
(8)where 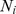 is the total number of misincorporations at nucleotide position *i* summed across all templates, *L* is the length of the template in bases, and 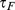 is the final time of recording. Our cost function penalizes the difference between the actual number of misincorporations on a nucleotide and the expected number of misincorporations (at the concentration being queried). When the concentration trace is expected to be sparse and/or smooth, additional terms, for example 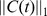 and/or 
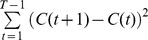
 respectively, can be included in the cost function. We optimize this cost function using a constrained gradient-based solver based on line-search methods, *“minConf,”* [53].

### Estimation of binary time-varying concentrations

To estimate an original binary time-varying ion concentration, we run the forward model with many different binary time-varying ion concentrations and determine which is the most likely to produce the observed sequences. Assuming independent binary (Bernoulli) events, the likelihood that a concentration will result in the observed sequences is given by the binomial distribution [52]:
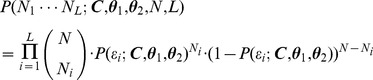
(9)


Limiting the acceptable input concentrations to “high” and “low” values (1 and 0) turns the concentration vector into a bit sequence. Thus, to estimate the sequence of *k* concentration pulses, or bits, we have to search in a binary space of dimensionality *k*. In our simulations, we limit the number of bits to 10, as accuracy does not significantly degrade beyond this number of bits for many relevant parameter values, and this allows a full exploration of the binary space.

### DNAP parameter estimation

To estimate the DNAP parameters from sequencing data, we look for the parameters that are most likely to result in the observed sequences, using *Eq. 9*. We search for the DNAP parameters 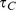, 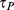, and 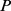 (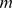 and 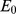 are assumed to be known from previous experiments) that give the highest likelihood using the Nelder-Mead SIMPLEX algorithm [54]. The fixed, time-varying ion concentration signal that we use to estimate the DNAP parameter values affects the estimation accuracy, and we therefore initially test parameter estimation at several time-varying concentrations. The concentration trace that allows the most accurate parameter estimation (during testing with known parameters) is then used for all parameter estimations in this study.

### Testing estimation accuracy

We used simulated molecular recording experiments to test the accuracy of ion concentration estimation using recording time resolutions and durations relevant to neural recording experiments. We determined the DNAP kinetic parameters, CMLF, and number of DNA templates required for various neural applications, and how they are affected by DNAP dissociation from the template, start-time variation, and initially unknown DNAP kinetic parameters. DNAP dissociation was considered by adding in an additional exponential to the dwell time distribution (*Eq. 2*), and start-time variation was considered by convolving a Gamma distribution representing varying start-times with the time distribution of nucleotide incorporations (like in *Eq. 3*). Details of our simulation methods can be found in *Text S5*.

## Supporting Information

Figure S1
**Optimality of continuous ion concentration estimation.** For a single ion concentration pulse, the variance of ion concentration estimation using our Fisher information framework (red) is compared to estimation accuracy computed using our unconstrained (green) and constrained (blue) continuous decoding algorithms on simulated data. As the variance derived from the Fisher information framework (Cramer-Rao bound: 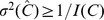) assumes unbiased estimation, the red and green curves are comparable. Estimation constraints provide additional information that can be used to further reduce the variance (of the blue curve). These plots use a concentration of 0.5, and similar plots exist for other concentrations. Experimental parameters are set as: 20 minutes of recording, 150 second concentration pulse, 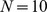, 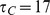 ms, 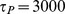 ms, 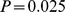, 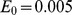, and 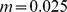 (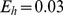). Error bars are standard errors of the mean accuracy, produced by bootstrapping. In each panel, one parameter is allowed to vary: **A**) the number of DNA templates, *N*, **B**) the baseline misincorporation rate, 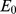 , **C**) the slope of the CMLF, *m*, **D**) the pause frequency, *P*, **E**) the duration of concentration pulses, and **F**) the end time of the pulse.(TIF)

Figure S2
**Optimality of binary ion concentration estimation.** For a single ion concentration pulse, the approximate decoding accuracy derived from our information-theoretic framework (red) is compared to decoding accuracy computed using simulations of our binary decoding algorithm (blue). Experimental parameters are set as: 20 minutes of recording, 15 second concentration pulse, 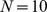, 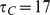 ms, 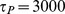 ms, 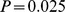, 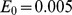, and 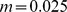 (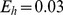). Error bars are standard errors of the mean accuracy produced by bootstrapping. In all panels (**A–F**), one parameter is allowed to vary as is described in the legend of Fig. S1.(TIF)

Figure S3
**DNAP dissociation from the template.** The error of ion concentration estimation is shown for varying re-association times for DNAPs with processivities of 1000 (blue) and 100 (red) in a multi-condition experiment. Solid lines are median estimation errors, and dashed lines are 95% confidence intervals. Used parameters are: 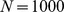, 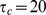 ms, 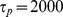 ms, *P* = 0.05, 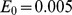 , and 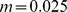 (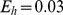).(TIF)

Figure S4 **Concentration fluctuations.** Estimation error for concentrations that are fixed (blue) and allowed to fluctuate (red) during each condition in a multi-condition experiment, as a function of number of templates. When estimating fixed concentrations, the concentrations at each condition are confined to be 0.2 to 0.8 (estimated values can still be between 0 and 1). When estimating fluctuating concentrations, the “baseline” concentration at each condition is also confined between 0.2 and 0.8, but the concentration value at every ms is chosen randomly from the interval [baseline-0.2 baseline+0.2]. For the fluctuation condition, we are attempting to estimate the mean concentration for each condition. Solid lines are median estimation errors, and dashed lines are 95% confidence intervals.
(TIF)

Figure S5
**Varying numbers of presented conditions.** **A**) Ion concentration estimation accuracy as a function of the number of different conditions tested within a 20 minute experiment. Solid lines are median estimation errors, and dashed lines are 95% confidence intervals. 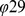 DNAP kinetic parameters, *N* = 1000, 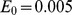, and 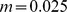 are used. **B**) For an experiment with 32 conditions, the median ion concentration estimation error with varying DNAP pausing parameters, a set elongation time of 5 ms, and the same additional parameters as panel A. Note that the scale differs from that of Fig. 3.(TIF)

Figure S6
**Effects of polymerase parameters on misincorporation probabilities.** **A**) Different combinations of the three DNAP kinetic parameters. **B**) The time distribution for the addition of the 50th nucleotide, 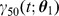 for each set of parameter values. **C**) An example time-varying concentration, used to calculate the misincorporation probabilities shown in panel D. The CMLF is set as 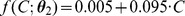. **D**) The misincorporation probability for the 50^th^ nucleotide, for the three simulations with different parameter combinations.(TIF)

Text S1 **Fisher information.** Further simplifications of the Fisher information equations in several limits, discussion of optimal concentration estimation given the derived Cramer Rao Bound, Fisher information with respect to additional pulse properties, and full derivations.
(PDF)

Text S2 **Multi-condition experiments.** Further details about the results of multi-condition experiments when varying the number of templates, CMLFs, DNAP kinetic parameters, and number of conditions, and considering the effects of dissociation and asynchronous start-times.
(PDF)

Text S3 **Importance of DNAP parameters.** Discussion regarding how DNAP parameters affect the distribution of times at which nucleotides are written, and how this alters the resulting misincorporation probabilities.
(PDF)

Text S4 **DNAP model.** A general discussion about DNAP dwell time distributions and the assumptions made to produce our simplified model.
(PDF)

Text S5 **Methods** **for testing simulations.** Details are given regarding how all simulations are run.
(PDF)

## References

[1] Church GM, Shendure J (2003) Nucleic Acid Memory Device. US Patent 20030228611.

[2] Kording KP (2011) Of toasters and molecular ticker tapes. PLoS Comput Biol 7: e1002291. 22219716 10.1371/journal.pcbi.1002291 PMC3248391

[3] El-Deiry WS, Downey KM, So AG (1984) Molecular mechanisms of manganese mutagenesis. Proc Natl Acad Sci U S A 81: 7378–7382. 6095289 10.1073/pnas.81.23.7378 PMC392149

[4] Zamft BM, Marblestone AH, Kording K, Schmidt D, Martin-Alarcon D, et al (2012) Measuring Cation Dependent DNA Polymerase Fidelity Landscapes by Deep Sequencing. PLoS One 7: e43876. 22928047 10.1371/journal.pone.0043876 PMC3425509

[5] Viguera E, Canceill D, Ehrlich SD (2001) Replication slippage involves DNA polymerase pausing and dissociation. EMBO J 20: 2587–2595. 11350948 10.1093/emboj/20.10.2587 PMC125466

[6] Schwartz JJ, Quake SR (2009) Single molecule measurement of the “speed limit” of DNA polymerase. Proc Natl Acad Sci U S A 106: 20294–20299. 19906998 10.1073/pnas.0907404106 PMC2787106

[7] Moffitt JR, Chemla YR, Bustamante C (2010) Methods in statistical kinetics. Methods Enzymol 475: 221–257. 20627160 10.1016/S0076-6879(10)75010-2

[8] Smith TF, Waterman MS (1981) Identification of common molecular subsequences. J Mol Biol 147: 195–197. 7265238 10.1016/0022-2836(81)90087-5

[9] Cramér H (1946) Methods of mathematical statistics. Princeton: Princeton University Press. 575 p.

[10] Miura K (2011) An Introduction to Maximum Likelihood Estimation and Information Geometry. Interdisciplinary Information Sciences 17: 155–174.

[11] Koch C (1999) Biophysics of computation: information processing in single neurons. New York: Oxford University Press. 562 p.

[12] Ibarra B, Chemla YR, Plyasunov S, Smith SB, Lazaro JM, et al (2009) Proofreading dynamics of a processive DNA polymerase. EMBO J 28: 2794–2802. 19661923 10.1038/emboj.2009.219 PMC2750014

[13] Ahrens MB, Orger MB, Robson DN, Li JM, Keller PJ (2013) Whole-brain functional imaging at cellular resolution using light-sheet microscopy. Nat Meth 10: 413–420. 10.1038/nmeth.2434 23524393

[14] Kelman Z, O'Donnell M (1995) DNA polymerase III holoenzyme: structure and function of a chromosomal replicating machine. Annu Rev Biochem 64: 171–200. 7574479 10.1146/annurev.bi.64.070195.001131

[15] Thomen P, Lopez PJ, Bockelmann U, Guillerez J, Dreyfus M, et al (2008) T7 RNA polymerase studied by force measurements varying cofactor concentration. Biophys J 95: 2423–2433. 18708471 10.1529/biophysj.107.125096 PMC2517023

[16] Skinner GM, Baumann CG, Quinn DM, Molloy JE, Hoggett JG (2004) Promoter binding, initiation, and elongation by bacteriophage T7 RNA polymerase. A single-molecule view of the transcription cycle. J Biol Chem 279: 3239–3244. 14597619 10.1074/jbc.M310471200

[17] Gauthier MG, Bechhoefer J (2009) Control of DNA replication by anomalous reaction-diffusion kinetics. Physical review letters 102: 158104. 19518676 10.1103/PhysRevLett.102.158104

[18] Frank EG, Woodgate R (2007) Increased catalytic activity and altered fidelity of human DNA polymerase iota in the presence of manganese. J Biol Chem 282: 24689–24696. 17609217 10.1074/jbc.M702159200

[19] Sanchez-Vives MV, McCormick DA (2000) Cellular and network mechanisms of rhythmic recurrent activity in neocortex. Nat Neurosci 3: 1027–1034. 11017176 10.1038/79848

[20] Ward LM (2003) Synchronous neural oscillations and cognitive processes. Trends Cogn Sci 7: 553–559. 14643372 10.1016/j.tics.2003.10.012

[21] Engel AK, Konig P, Kreiter AK, Schillen TB, Singer W (1992) Temporal coding in the visual cortex: new vistas on integration in the nervous system. Trends Neurosci 15: 218–226. 1378666 10.1016/0166-2236(92)90039-b

[22] Sokoloff L (1981) Localization of functional activity in the central nervous system by measurement of glucose utilization with radioactive deoxyglucose. J Cereb Blood Flow Metab 1: 7–36. 7035471 10.1038/jcbfm.1981.4

[23] Friedman HR, Bruce CJ, Goldmanrakic PS (1989) Resolution of Metabolic Columns by a Double-Label 2-Dg Technique - Interdigitation and Coincidence in Visual Cortical Areas of the Same Monkey. Journal of Neuroscience 9: 4111–4121. 2687438 10.1523/JNEUROSCI.09-12-04111.1989 PMC6569629

[24] Langhorst BW, Jack WE, Reha-Krantz L, Nichols NM (2012) Polbase: a repository of biochemical, genetic and structural information about DNA polymerases. Nucleic Acids Res 40: D381–D387. 21993301 10.1093/nar/gkr847 PMC3245023

[25] Davenport RJ, Wuite GJ, Landick R, Bustamante C (2000) Single-molecule study of transcriptional pausing and arrest by E. coli RNA polymerase. Science 287: 2497–2500. 10741971 10.1126/science.287.5462.2497

[26] Mejia YX, Mao H, Forde NR, Bustamante C (2008) Thermal probing of E. coli RNA polymerase off-pathway mechanisms. J Mol Biol 382: 628–637. 18647607 10.1016/j.jmb.2008.06.079 PMC2615098

[27] Hodges C, Bintu L, Lubkowska L, Kashlev M, Bustamante C (2009) Nucleosomal fluctuations govern the transcription dynamics of RNA polymerase II. Science 325: 626–628. 19644123 10.1126/science.1172926 PMC2775800

[28] Zamft B, Bintu L, Ishibashi T, Bustamante C (2012) Nascent RNA structure modulates the transcriptional dynamics of RNA polymerases. Proc Natl Acad Sci U S A 109: 8948–8953. 22615360 10.1073/pnas.1205063109 PMC3384149

[29] Vogelstein JT, Packer AM, Machado TA, Sippy T, Babadi B, et al (2010) Fast nonnegative deconvolution for spike train inference from population calcium imaging. J Neurophysiol 104: 3691–3704. 20554834 10.1152/jn.01073.2009 PMC3007657

[30] Vogelstein JT, Watson BO, Packer AM, Yuste R, Jedynak B, et al (2009) Spike Inference from Calcium Imaging Using Sequential Monte Carlo Methods. Biophys J 97: 636–655. 19619479 10.1016/j.bpj.2008.08.005 PMC2711341

[31] Mishchenko Y, Vogelstein JT, Paninski L (2011) A Bayesian Approach for Inferring Neuronal Connectivity from Calcium Fluorescent Imaging Data. Annals of Applied Statistics 5: 1229–1261.

[32] Yuste R, MacLean J, Vogelstein J, Paninski L (2011) Imaging action potentials with calcium indicators. Cold Spring Harb Protoc 2011: 985–989. 21807854 10.1101/pdb.prot5650

[33] Chun M, Church GM, Greenspan RJ, Roukes ML, Yuste R (2012) The Brain Activity Map Project and the Challenge of Functional Connectomics. Neuron 74: 970–974. 22726828 10.1016/j.neuron.2012.06.006 PMC3597383

[34] Hays H, Berdis AJ (2002) Manganese substantially alters the dynamics of translesion DNA synthesis. Biochemistry 41: 4771–4778. 11939771 10.1021/bi0120648

[35] Dempster AP, Laird NM, Rubin DB (1977) Maximum Likelihood from Incomplete Data via the EM Algorithm. Journal of the Royal Statistical Society Series B (Methodological) 39: 1–38.

[36] Cornish-Bowden A (1979) Fundamentals of enzyme kinetics. London; Boston: Butterworths. xiii, 230 p. p.

[37] Sharma AK, Chowdhury D (2012) Error correction during DNA replication. Physical Review E 86: 011913. 10.1103/PhysRevE.86.011913 23005458

[38] Shaevitz JW, Block SM, Schnitzer MJ (2005) Statistical kinetics of macromolecular dynamics. Biophys J 89: 2277–2285. 16040752 10.1529/biophysj.105.064295 PMC1366729

[39] Chemla YR, Moffitt JR, Bustamante C (2008) Exact solutions for kinetic models of macromolecular dynamics. J Phys Chem B 112: 6025–6044. 18373360 10.1021/jp076153r

[40] Kolomeisky AB, Fisher ME (2007) Molecular Motors: A Theorist's Perspective. Annual Review of Physical Chemistry 58: 675–695. 10.1146/annurev.physchem.58.032806.104532 17163836

[41] Bell AJ, Sejnowski TJ (1995) An information-maximization approach to blind separation and blind deconvolution. Neural Comput 7: 1129–1159. 7584893 10.1162/neco.1995.7.6.1129

[42] Kirkeby O, Nelson PA, Hamada H, Orduna-Bustamante F (1998) Fast deconvolution of multichannel systems using regularization (Reprinted from IEICE Transactions on Fundamentals of Electronics, Communications, and Computer Sciences, vol E80-A, pg 809–820, 1997). Ieee Transactions on Speech and Audio Processing 6: 189–194.

[43] Yu B, Afshar A, Santhanam G, Ryu SI, Shenoy K, et al (2006) Extracting dynamical structure embedded in neural activity. Advances in Neural Information Processing Systems 18: 1545.

[44] Macke JH, Buesing L, Cunningham JP, Yu BM, Shenoy KV, et al (2011) Empirical models of spiking in neural populations. Advances in Neural Information Processing Systems 24: 13501358.

[45] Sahani M, Linden JF (2003) Evidence Optimization Techniques for Estimating Stimulus-Response Functions. Advances in Neural Information Processing Systems 301–308.

[46] Moffitt JR, Chemla YR, Bustamante C (2010) Mechanistic constraints from the substrate concentration dependence of enzymatic fluctuations. Proc Natl Acad Sci U S A 107: 15739–15744. 20729471 10.1073/pnas.1006997107 PMC2936640

[47] Moffitt JR, Chemla YR, Aathavan K, Grimes S, Jardine PJ, et al (2009) Intersubunit coordination in a homomeric ring ATPase. Nature 457: 446–450. 19129763 10.1038/nature07637 PMC2716090

[48] Wang J, Wagner F, Borton DA, Zhang J, Ozden I, et al (2012) Integrated device for combined optical neuromodulation and electrical recording for chronic in vivo applications. J Neural Eng 9: 016001. 22156042 10.1088/1741-2560/9/1/016001

[49] Buzsaki G (2004) Large-scale recording of neuronal ensembles. Nat Neurosci 7: 446–451. 15114356 10.1038/nn1233

[50] Carr PA, Church GM (2009) Genome engineering. Nat Biotechnol 27: 1151–1162. 20010598 10.1038/nbt.1590

[51] Morin JA, Cao FJ, Lazaro JM, Arias-GonzaIez JR, Valpuesta JM, et al (2012) Active DNA unwinding dynamics during processive DNA replication. Proc Natl Acad Sci U S A 109: 8115–8120. 22573817 10.1073/pnas.1204759109 PMC3361432

[52] Hogg RV, McKean JW, Craig AT (2005) Introduction to mathematical statistics. Upper Saddle River, N.J.: Pearson Education. xiii, 704 p. p.

[53] Schmidt M (2008) minConf. Available: http://www.di.ens.fr/~mschmidt/Software/minFunc.html. Accessed August 2012.

[54] Nelder JA, Mead R (1965) A Simplex-Method for Function Minimization. Computer Journal 7: 308–313.

