# Supplementary figures and images for: Statistical Analysis of Molecular Signal Recording
Source: PLoS Comput Biol. 2013 Jul 18;9(7):e1003145. doi: 10.1371/journal.pcbi.1003145 (PMC3715445; doi:10.1371/journal.pcbi.1003145)

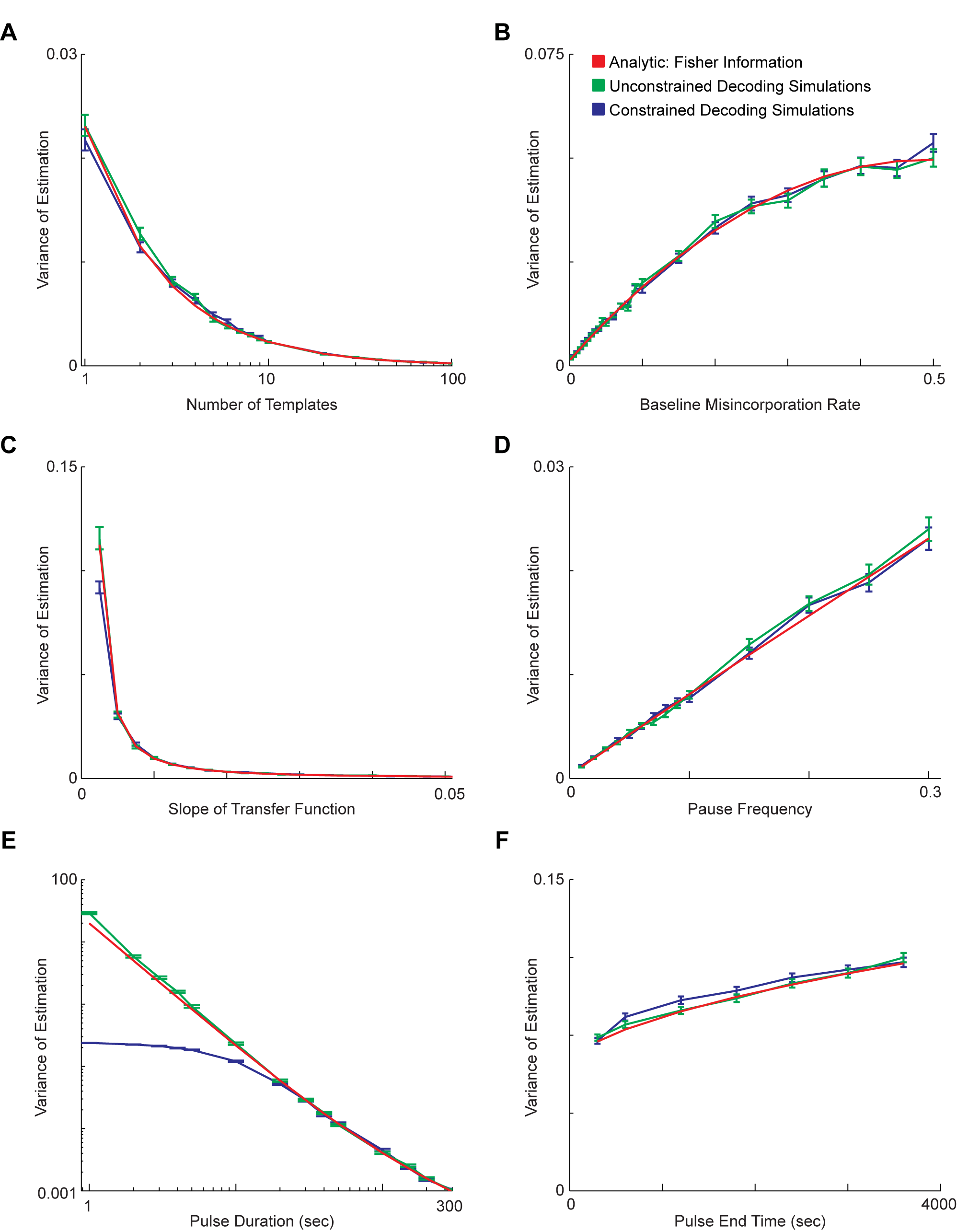

Supplement: Figure S1 — Optimality of continuous ion concentration estimation. For a single ion concentration pulse, the variance of ion concentration estimation using our Fisher information framework (red) is compared to estimation accuracy computed using our unconstrained (green) and constrained (blue) continuous decoding algorithms on simulated data. As the variance derived from the Fisher information framework (Cramer-Rao bound: ) assumes unbiased estimation, the red and green curves are comparable. Estimation constraints provide additional information that can be used to further reduce the variance (of the blue curve). These plots use a concentration of 0.5, and similar plots exist for other concentrations. Experimental parameters are set as: 20 minutes of recording, 150 second concentration pulse, , ms, ms, , , and (). Error bars are standard errors of the mean accuracy, produced by bootstrapping. In each panel, one parameter is allowed to vary: A) the number of DNA templates, N, B) the baseline misincorporation rate, , C) the slope of the CMLF, m, D) the pause frequency, P, E) the duration of concentration pulses, and F) the end time of the pulse. (TIF) [file pcbi.1003145.s001.tif]

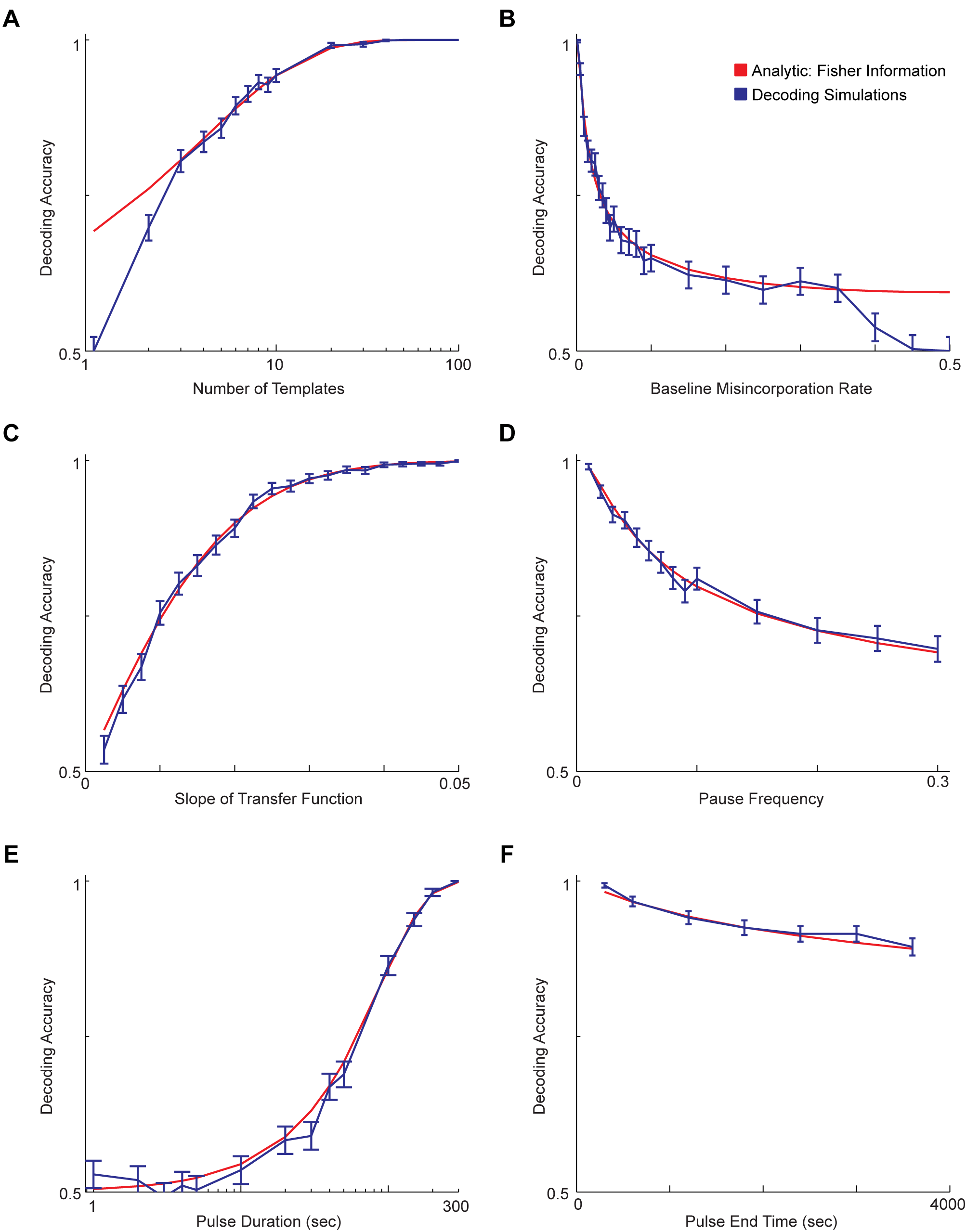

Supplement: Figure S2 — Optimality of binary ion concentration estimation. For a single ion concentration pulse, the approximate decoding accuracy derived from our information-theoretic framework (red) is compared to decoding accuracy computed using simulations of our binary decoding algorithm (blue). Experimental parameters are set as: 20 minutes of recording, 15 second concentration pulse, , ms, ms, , , and (). Error bars are standard errors of the mean accuracy produced by bootstrapping. In all panels (A–F), one parameter is allowed to vary as is described in the legend of Fig. S1. (TIF) [file pcbi.1003145.s002.tif]

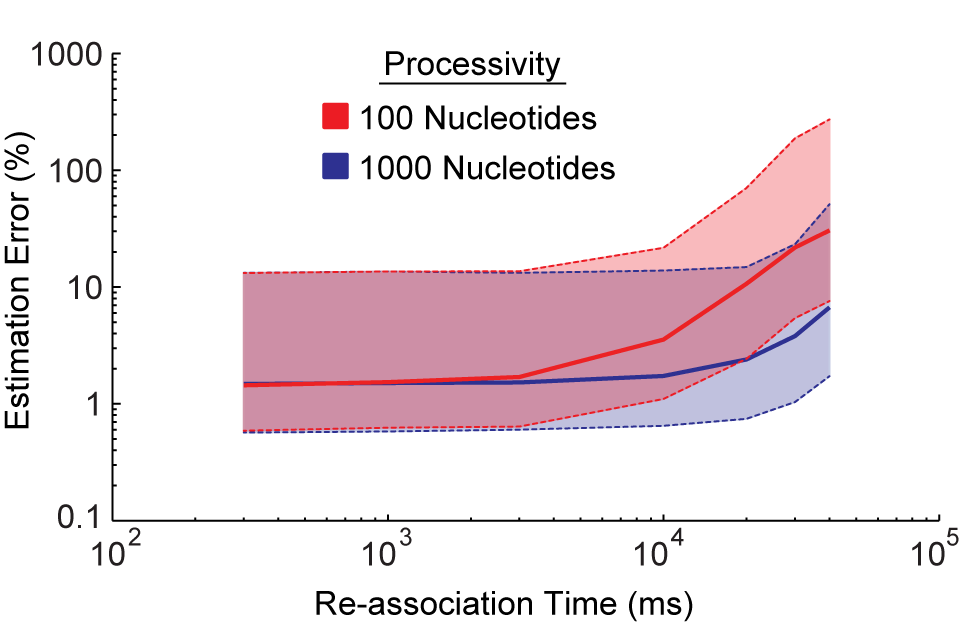

Supplement: Figure S3 — DNAP dissociation from the template. The error of ion concentration estimation is shown for varying re-association times for DNAPs with processivities of 1000 (blue) and 100 (red) in a multi-condition experiment. Solid lines are median estimation errors, and dashed lines are 95% confidence intervals. Used parameters are: , ms, ms, P = 0.05, , and (). (TIF) [file pcbi.1003145.s003.tif]

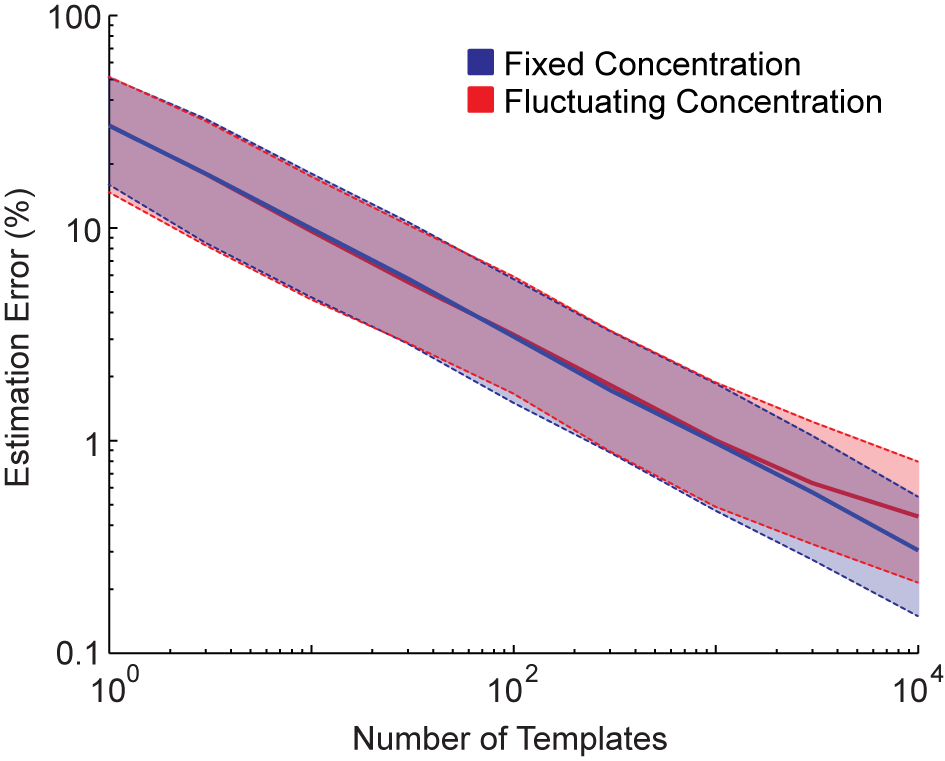

Supplement: Figure S4 — Concentration fluctuations. Estimation error for concentrations that are fixed (blue) and allowed to fluctuate (red) during each condition in a multi-condition experiment, as a function of number of templates. When estimating fixed concentrations, the concentrations at each condition are confined to be 0.2 to 0.8 (estimated values can still be between 0 and 1). When estimating fluctuating concentrations, the “baseline” concentration at each condition is also confined between 0.2 and 0.8, but the concentration value at every ms is chosen randomly from the interval [baseline-0.2 baseline+0.2]. For the fluctuation condition, we are attempting to estimate the mean concentration for each condition. Solid lines are median estimation errors, and dashed lines are 95% confidence intervals. (TIF) [file pcbi.1003145.s004.tif]

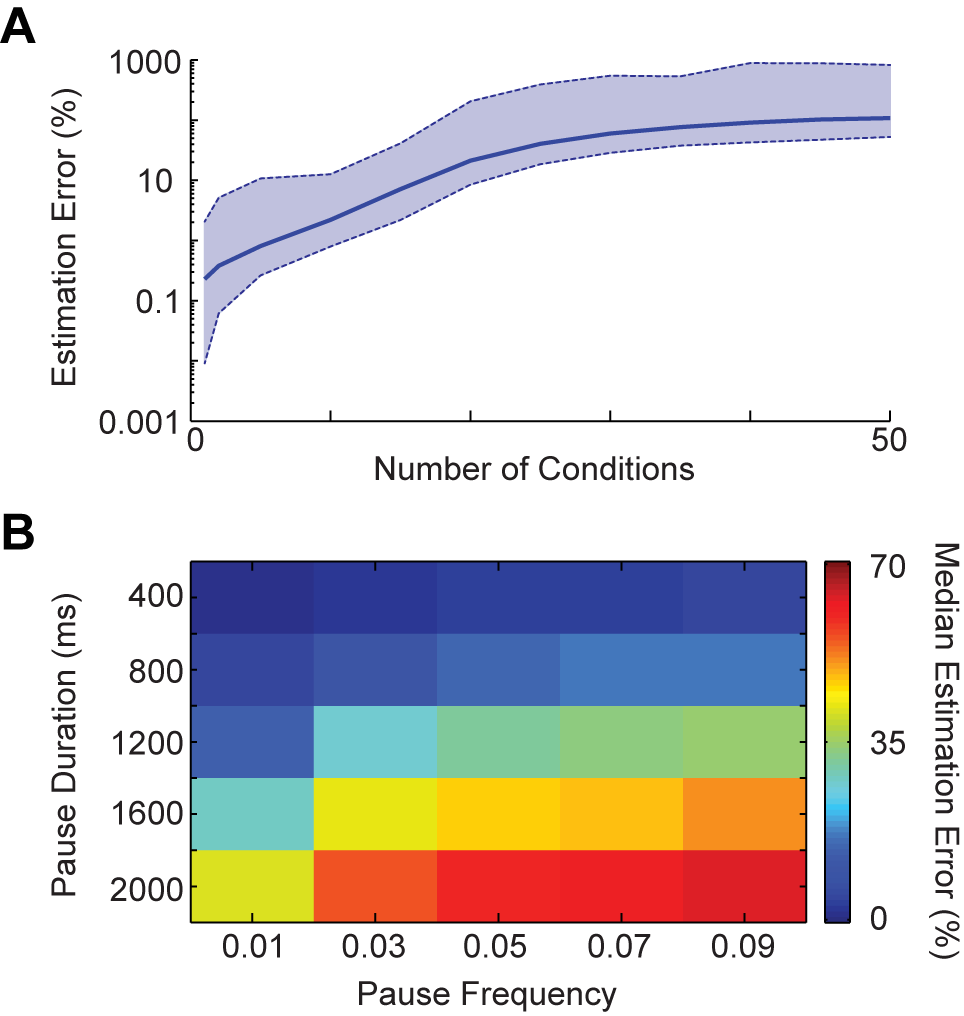

Supplement: Figure S5 — Varying numbers of presented conditions. A) Ion concentration estimation accuracy as a function of the number of different conditions tested within a 20 minute experiment. Solid lines are median estimation errors, and dashed lines are 95% confidence intervals. DNAP kinetic parameters, N = 1000, , and are used. B) For an experiment with 32 conditions, the median ion concentration estimation error with varying DNAP pausing parameters, a set elongation time of 5 ms, and the same additional parameters as panel A. Note that the scale differs from that of Fig. 3. (TIF) [file pcbi.1003145.s005.tif]

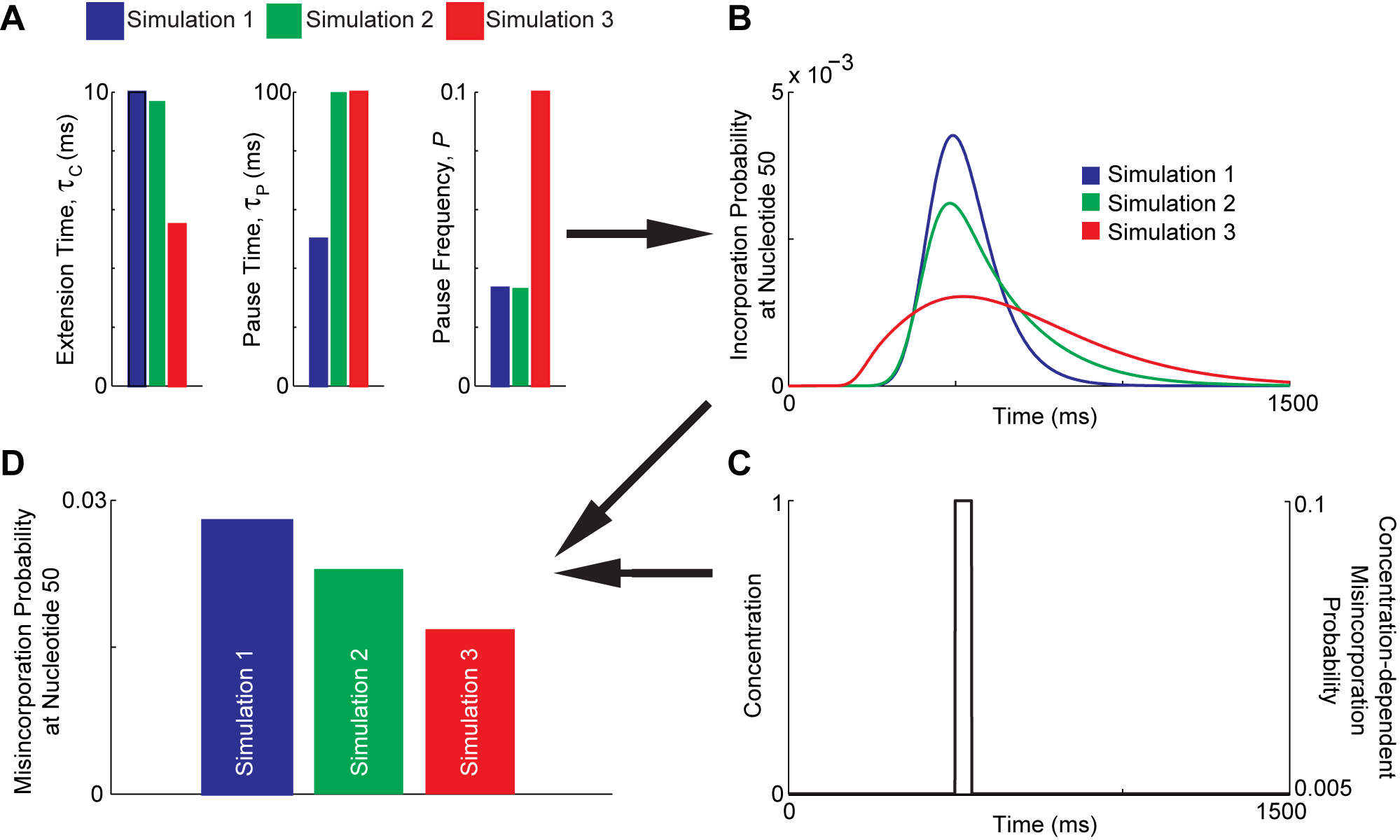

Supplement: Figure S6 — Effects of polymerase parameters on misincorporation probabilities. A) Different combinations of the three DNAP kinetic parameters. B) The time distribution for the addition of the 50th nucleotide, for each set of parameter values. C) An example time-varying concentration, used to calculate the misincorporation probabilities shown in panel D. The CMLF is set as . D) The misincorporation probability for the 50th nucleotide, for the three simulations with different parameter combinations. (TIF) [file pcbi.1003145.s006.tif]
