# Supplementary material for: Statistical Analysis of Molecular Signal Recording
Source: PLoS Comput Biol. 2013 Jul 18;9(7):e1003145. doi: 10.1371/journal.pcbi.1003145 (PMC3715445; doi:10.1371/journal.pcbi.1003145)
Supplement: Text S1 — Fisher information. Further simplifications of the Fisher information equations in several limits, discussion of optimal concentration estimation given the derived Cramer Rao Bound, Fisher information with respect to additional pulse properties, and full derivations. (PDF) [file pcbi.1003145.s007.pdf]

Text S1. Fisher information

Further simplifications

We can further simplify *Eq. 1* by imposing additional limits beyond a small overall misincorporation rate (Note that $\Gamma_i(T_0,\delta;\boldsymbol{\theta}_1)$ is written as Γ_i for brevity, as is done in *Methods*). In the limit of high relative baseline misincorporation rates ($E_0 >> m \cdot \Gamma_i \cdot C$), or in the limit of small concentrations ($C << \frac{E_0}{m \cdot \Gamma_i}$), $I(C)_{N\_templates} \propto \sum_i \left(m \cdot \Gamma_i\right)^2$. In the opposite limits ($E_0 << m \cdot \Gamma_i \cdot C$ or $C >> \frac{E_0}{m \cdot \Gamma_i}$), $I(C)_{N\_templates} \propto \sum_i m \cdot \Gamma_i$. Thus, the baseline misincorporation rate and the height of the concentration pulse affect how the Fisher information scales with *m* and Γ_i. Compensating for a larger baseline misincorporation rate or for a small peak concentration requires a larger misincorporation rate increase per unit of ion concentration change (CMLF slope, *m*), a longer pulse (corresponding to lower achievable time resolution), an earlier pulse start-time (to limit the degree of de-phasing between members of the polymerase ensemble), or less stochastic DNAP dynamics.

Optimal continuous concentration estimation

Using *Eq. 7c*, we treat the inverse problem of analytically determining how the accuracy of estimating a single ion concentration pulse depends on the polymerase parameters, the length of the template, and the duration of the ion pulse

(i.e. temporal resolution). We continue to consider the simplified case with a single ion concentration step. The Cramer-Rao bound (CRB) states that the minimum variance of an unbiased estimator of a parameter θ is the reciprocal of the Fisher information $I(\theta)$ [9]:

$$\sigma^2(\hat{C}) \geq \left(N \sum_i \frac{\left(m \cdot \Gamma_i\right)^2}{\left(E_0 + m \cdot \Gamma_i \cdot C\right) \cdot \left(1 - E_0 - m \cdot \Gamma_i \cdot C\right)} \right)^{-1} \tag{S1}$$

The CRB applies only to unbiased estimators. Our estimator is biased, as we constrain the concentration to be between zero and one, and thus the CRB does not represent the theoretical minimum variance of our estimator. In other words, our constrained estimation algorithm can theoretically perform better than *Eq. S1*. Thus, in Fig. S1, we also compare the CRB to results from unconstrained estimation of concentrations.

Optimal binary decoding accuracy

We use the Cramer-Rao bound to approximate the theoretical optimal accuracy of binary concentration decoding (determining if $C = 0$ or $C = 1$). To do this, we first calculate the minimum estimation variance (CRB) at parameter values $C = 0$ and $C = 1$. We next define two Gaussian distributions with known means (0 and 1) and variances that represent the theoretical distributions of estimator values when optimally estimating the concentrations 0 and 1. In order to determine the theoretical minimum error of binary estimation, we ask how likely it is that a value drawn from one Gaussian distribution could be mistaken to come from the other Gaussian distribution. We approximate this by setting a "decision threshold"

between the two distributions, where values of C below the threshold should be classified as a low concentration, and values of C above this threshold should be classified as a high concentration. Thus, the probability that the Gaussian centered on C=1 is below this threshold is the probability of false negatives (claiming C=0 when C=1). The probability of false positives (claiming C=1 when C=0) is found in the same manner. We set the threshold as the location where the two probability density functions are equal. We consider the decoding error as the average probability of false positives and false negatives, assuming an equal prior for low and high concentrations (as we do in our simulations). The decoding accuracy is defined as one minus this decoding error.

Additional pulse properties

In the main text, we focus on the ability to estimate an ion concentration at a given time (or time interval), assuming that both the start-time and duration of the concentration pulse are known. This is a reasonable assumption in the case of determining neural responses to known stimuli (i.e. multi-condition experiments), which we focus on in the main text. It also would not interfere with determining slow synchronizations, as we only want to determine whether two neurons fired together within a known time segment. However, more generally, one might want to know the ability of molecular ticker tapes to predict the start-time, T_0, and duration of a pulse, δ. Thus, in the simplified case of a single pulse, we show the Fisher information contained in nucleotide misincorporations of these additional pulse properties.

To calculate these Fisher information expressions, we make the unrealistic assumption that all other parameters are fixed, so that the provided expressions are simple and can help in providing intuition. One would need a 3×3 matrix to determine Fisher information while allowing the concentration, pulse start-time, and pulse duration to vary.

The Fisher information with respect to pulse start-time is:

$$I(T_0)_{Nucleotide\_i} = E\left[\left(\frac{\partial}{\partial T_0}\log\left(g\left(X_i;T_0\right)\right)\right)^2\right] = \frac{\left(m\cdot C\cdot\left(\gamma_i(T_0+\delta;\boldsymbol{\theta}_1)-\gamma_i(T_0;\boldsymbol{\theta}_1)\right)\right)^2}{\left(E_0+m\cdot\Gamma_i\cdot C\right)\left(1-E_0-m\cdot\Gamma_i\cdot C\right)} \qquad \text{(S2a)}$$

We assume that misincorporation probabilities at successive template bases are approximately independent, and that individual templates are copied independently, so that:

$$I(T_0)_{N\_templates} = N\sum_i \frac{\left(m\cdot C\cdot\left(\gamma_i(T_0+\delta;\boldsymbol{\theta}_1)-\gamma_i(T_0;\boldsymbol{\theta}_1)\right)\right)^2}{\left(E_0+m\cdot\Gamma_i\cdot C\right)\left(1-E_0-m\cdot\Gamma_i\cdot C\right)} \qquad \text{(S2b)}$$

The Fisher information with respect to the pulse duration is:

$$I(\delta)_{Nucleotide\_i} = E\left[\left(\frac{\partial}{\partial \delta}\log\left(g\left(X_i;\delta\right)\right)\right)^2\right] = \frac{\left(m\cdot C\cdot\gamma_i(T_0+\delta;\boldsymbol{\theta}_1)\right)^2}{\left(E_0+m\cdot\Gamma_i\cdot C\right)\left(1-E_0-m\cdot\Gamma_i\cdot C\right)} \qquad \text{(S3a)}$$

We assume that misincorporation probabilities at successive template bases are approximately independent, and that individual templates are copied independently, so that:

$$I(\delta)_{N\_templates} = N\sum_i \frac{\left(m\cdot C\cdot\gamma_i(T_0+\delta;\boldsymbol{\theta}_1)\right)^2}{\left(E_0+m\cdot\Gamma_i\cdot C\right)\left(1-E_0-m\cdot\Gamma_i\cdot C\right)} \qquad \text{(S3b)}$$

Derivations: pulse magnitude (concentration)

$$g\left(X_i;C\right)=\left(E_0+m\cdot\Gamma_i\cdot C\right)\cdot\left(1-X_i\right)+\left(1-E_0-m\cdot\Gamma_i\cdot C\right)\cdot\left(X_i\right)$$

$$I(C)_{Nucleotide\_i}=E\left[\left(\frac{\partial}{\partial C}\log\left(g\left(X_i;C\right)\right)\right)^2\right]$$

$$=\left[\left(\frac{\partial}{\partial C}\log\left(g\left(X_i=0;C\right)\right)\right)^2\right]\left[g\left(X_i=0;C\right)\right]+\left[\left(\frac{\partial}{\partial C}\log\left(g\left(X_i=1;C\right)\right)\right)^2\right]\left[g\left(X_i=1;C\right)\right]$$

$$=\left(\frac{\partial}{\partial C}\log\left(E_0+m\cdot\Gamma_i\cdot C\right)\right)^2\left(E_0+m\cdot\Gamma_i\cdot C\right)+\left(\frac{\partial}{\partial C}\log\left(1-E_0-m\cdot\Gamma_i\cdot C\right)\right)^2\left(1-E_0-m\cdot\Gamma_i\cdot C\right)$$

$$=\left(\frac{m\cdot\Gamma_i}{E_0+m\cdot\Gamma_i\cdot C}\right)^2\left(E_0+m\cdot\Gamma_i\cdot C\right)+\left(\frac{-m\cdot\Gamma_i}{1-E_0-m\cdot\Gamma_i\cdot C}\right)^2\left(1-E_0-m\cdot\Gamma_i\cdot C\right)$$

$$=\frac{\left(m\cdot\Gamma_i\right)^2}{E_0+m\cdot\Gamma_i\cdot C}+\frac{\left(m\cdot\Gamma_i\right)^2}{1-E_0-m\cdot\Gamma_i\cdot C}$$

$$=\frac{\left(m\cdot\Gamma_i\right)^2}{\left(E_0+m\cdot\Gamma_i\cdot C\right)\left(1-E_0-m\cdot\Gamma_i\cdot C\right)}$$

Derivations: pulse start-time

$$g\left(X_i;T_0\right)=\left(E_0+m\cdot\Gamma_i\cdot C\right)\cdot\left(1-X_i\right)+\left(1-E_0-m\cdot\Gamma_i\cdot C\right)\cdot\left(X_i\right)$$

$$I(T_0)_{Nucleotide\_i}=E\left[\left(\frac{\partial}{\partial T_0}\log\left(g\left(X_i;T_0\right)\right)\right)^2\right]$$

$$=\left[\left(\frac{\partial}{\partial T_0}\log\left(g\left(X_i=0;T_0\right)\right)\right)^2\right]\left[g\left(X_i=0;T_0\right)\right]+\left[\left(\frac{\partial}{\partial T_0}\log\left(g\left(X_i=1;T_0\right)\right)\right)^2\right]\left[g\left(X_i=1;T_0\right)\right]$$

$$=\left(\frac{\partial}{\partial T_0}\log\left(E_0+m\cdot\Gamma_i\cdot C\right)\right)^2\left(E_0+m\cdot\Gamma_i\cdot C\right)+\left(\frac{\partial}{\partial T_0}\log\left(1-E_0-m\cdot\Gamma_i\cdot C\right)\right)^2\left(1-E_0-m\cdot\Gamma_i\cdot C\right)$$

$$\frac{\partial}{\partial T_0}\Gamma_i=\frac{\partial}{\partial T_0}\left[\int_{T_0}^{T_0+\delta}\gamma_i(t;\boldsymbol{\theta}_1)\,dt\right]=\gamma_i(T_0+\delta;\boldsymbol{\theta}_1)-\gamma_i(T_0;\boldsymbol{\theta}_1)$$

$$\frac{\partial}{\partial T_0}\log\left(E_0+m\cdot\Gamma_i\cdot C\right)=\frac{1}{E_0+m\cdot\Gamma_i\cdot C}\cdot\frac{\partial}{\partial T_0}\left[E_0+m\cdot\Gamma_i\cdot C\right]=\frac{m\cdot C\left(\gamma_i(T_0+\delta;\boldsymbol{\theta}_1)-\gamma_i(T_0;\boldsymbol{\theta}_1)\right)}{E_0+m\cdot\Gamma_i\cdot C}$$

$$I(T_0)_{Nucleotide\_i}=\left(\frac{m\cdot C\cdot\left(\gamma_i(T_0+\delta;\boldsymbol{\theta}_1)-\gamma_i(T_0;\boldsymbol{\theta}_1)\right)}{E_0+m\cdot\Gamma_i\cdot C}\right)^2\left(E_0+m\cdot\Gamma_i\cdot C\right)+\left(\frac{-m\cdot C\cdot\left(\gamma_i(T_0+\delta;\boldsymbol{\theta}_1)-\gamma_i(T_0;\boldsymbol{\theta}_1)\right)}{1-E_0-m\cdot\Gamma_i\cdot C}\right)^2\left(1-E_0-m\cdot\Gamma_i\cdot C\right)$$

$$=\frac{\left(m\cdot C\cdot\left(\gamma_i(T_0+\delta;\boldsymbol{\theta}_1)-\gamma_i(T_0;\boldsymbol{\theta}_1)\right)\right)^2}{E_0+m\cdot\Gamma_i\cdot C}+\frac{\left(m\cdot C\cdot\left(\gamma_i(T_0+\delta;\boldsymbol{\theta}_1)-\gamma_i(T_0;\boldsymbol{\theta}_1)\right)\right)^2}{1-E_0-m\cdot\Gamma_i\cdot C}$$

$$=\frac{\left(m\cdot C\cdot\left(\gamma_i(T_0+\delta;\boldsymbol{\theta}_1)-\gamma_i(T_0;\boldsymbol{\theta}_1)\right)\right)^2}{\left(E_0+m\cdot\Gamma_i\cdot C\right)\left(1-E_0-m\cdot\Gamma_i\cdot C\right)}$$

Derivations: pulse duration

$$g\left(X_i;\delta\right)=\left(E_0+m\cdot\Gamma_i\cdot C\right)\cdot\left(1-X_i\right)+\left(1-E_0-m\cdot\Gamma_i\cdot C\right)\cdot\left(X_i\right)$$

$$I(\delta)_{Nucleotide\_i}=E\left[\left(\frac{\partial}{\partial T_0}\log\left(g\left(X_i;\delta\right)\right)\right)^2\right]$$

$$=\left[\left(\frac{\partial}{\partial\delta}\log\left(g\left(X_i=0;\delta\right)\right)\right)^2\right]\left[g\left(X_i=0;\delta\right)\right]+\left[\left(\frac{\partial}{\partial\delta}\log\left(g\left(X_i=1;\delta\right)\right)\right)^2\right]\left[g\left(X_i=1;\delta\right)\right]$$

$$=\left(\frac{\partial}{\partial\delta}\log\left(E_0+m\cdot\Gamma_i\cdot C\right)\right)^2\left(E_0+m\cdot\Gamma_i\cdot C\right)+\left(\frac{\partial}{\partial\delta}\log\left(1-E_0-m\cdot\Gamma_i\cdot C\right)\right)^2\left(1-E_0-m\cdot\Gamma_i\cdot C\right)$$

$$\frac{\partial}{\partial\delta}\Gamma_i=\frac{\partial}{\partial\delta}\left[\int_{T_0}^{T_0+\delta}\gamma_i(t;\boldsymbol{\theta}_1)\,dt\right]=\gamma_i(T_0+\delta;\boldsymbol{\theta}_1)$$

$$\frac{\partial}{\partial\delta}\log\left(E_0+m\cdot\Gamma_i\cdot C\right)=\frac{1}{E_0+m\cdot\Gamma_i\cdot C}\cdot\frac{\partial}{\partial\delta}\left[E_0+m\cdot\Gamma_i\cdot C\right]=\frac{m\cdot C\cdot\gamma_i(T_0+\delta;\boldsymbol{\theta}_1)}{E_0+m\cdot\Gamma_i\cdot C}$$

$$I(\delta)_{Nucleotide}=\left(\frac{m\cdot C\cdot\gamma_i(T_0+\delta;\boldsymbol{\theta}_1)}{E_0+m\cdot\Gamma_i\cdot C}\right)^2\left(E_0+m\cdot\Gamma_i\cdot C\right)+\left(\frac{-m\cdot C\cdot\gamma_i(T_0+\delta;\boldsymbol{\theta}_1)}{1-E_0-m\cdot\Gamma_i\cdot C}\right)^2\left(1-E_0-m\cdot\Gamma_i\cdot C\right)$$

$$=\frac{\left(m\cdot C\cdot\gamma_i(T_0+\delta;\boldsymbol{\theta}_1)\right)^2}{E_0+m\cdot\Gamma_i\cdot C}+\frac{\left(m\cdot C\cdot\gamma_i(T_0+\delta;\boldsymbol{\theta}_1)\right)^2}{1-E_0-m\cdot\Gamma_i\cdot C}$$

$$=\frac{\left(m\cdot C\cdot\gamma_i(T_0+\delta;\boldsymbol{\theta}_1)\right)^2}{\left(E_0+m\cdot\Gamma_i\cdot C\right)\left(1-E_0-m\cdot\Gamma_i\cdot C\right)}$$
