# Supplementary material for: Statistical Analysis of Molecular Signal Recording
Source: PLoS Comput Biol. 2013 Jul 18;9(7):e1003145. doi: 10.1371/journal.pcbi.1003145 (PMC3715445; doi:10.1371/journal.pcbi.1003145)
Supplement: Text S2 — Multi-condition experiments. Further details about the results of multi-condition experiments when varying the number of templates, CMLFs, DNAP kinetic parameters, and number of conditions, and considering the effects of dissociation and asynchronous start-times. (PDF) [file pcbi.1003145.s008.pdf]

Text S2. Multi-condition experiments

We evaluated the feasibility of multi-condition experiments at varying experimental parameter values. Unless otherwise stated, the baseline parameter values, from which we varied one parameter at a time, were: 8 conditions, 20 minute experimental duration, φ29 DNAP kinetic parameters ($\tau_C \approx 17$ ms, $\tau_P \approx 3000$ ms, $P \approx 0.025$), $E_0 = 0.005$ and $m = 0.025$ ($E_h = 0.03$), and N=1000. We report the median estimation error because this statistic is robust to outliers.

Sensitivity to varying numbers of templates

We started by determining the ion concentration estimation error for varying numbers of DNA templates. With 1000 templates, the median estimation error was only 1.4%, (Fig. 3A). In realistic multi-condition experiments, the concentration may not actually be constant during each condition, as we have assumed above. However, minor concentration fluctuations during the duration of a single measurement condition do not have a significant effect on the estimation accuracy (Fig. S4). Therefore, high accuracy estimation of concentrations varying across several experimental conditions is possible using molecular recorders with a sufficient (and biologically plausible) number of templates.

Sensitivity to varying CMLFs

To test the role of the CMLF in estimation accuracy, we varied baseline misincorporation rates and CMLF slopes, while the number of templates was fixed

at 1000 (Fig. 3B). As expected, estimation error increases for larger baseline misincorporation rates (E_0), and decreases with greater CMLF slopes (*m*). Additionally, larger misincorporation rates are beneficial for a given $E_h : E_0$ ratio (recall that we set the maximum concentration as 1, so $E_h = E_0 + m$). For example, for $E_h = 0.2$ and $E_0 = 0.1$, there is only 1.1% median estimation error vs. 11.7% for $E_h = 0.002$ and $E_0 = 0.001$ (Fig. 3B). This pattern can also be seen theoretically from *Eq. 1.* This demonstrates that high fidelity decoding may be possible even with limited differences between misincorporation rates at low and high ion concentrations.

Sensitivity to DNAP kinetic parameters

We further explored the ranges of DNAP pausing parameters compatible with high accuracy ion concentration estimation, fixing the CMLF as $E_0 = 0.005$, $m = 0.025$ ($E_h = 0.03$), using 100 or 1000 DNA templates, and fixing the elongation time at 20 ms. With 1000 templates, highly stochastic parameters could be used, e.g., a pause duration of 3000 ms and pause frequency of 0.13 allowed estimation with only 3.2% median error (Fig. 3C). Using 100 templates, these pausing parameters yielded 9.4% median error, but this can be mitigated while remaining within realistic ranges for the DNAP pausing parameters (Fig. 3D). Therefore, a broad range of kinetic parameters within the range of documented DNAPs could be used in a molecular recording experiment to compare neural firing rates across several conditions.

Sensitivity to dissociation

In the previous sections, we have ignored the possibility of the DNAP dissociating from the template and being replaced by another polymerase molecule from solution. However, dissociation would be expected to introduce additional stochasticity into the movement of the polymerase and thus limit our ability to decode concentration signals. To address this concern, we test the effects of DNAP dissociation on ion concentration estimation accuracy in the context of an experiment with eight conditions. We first test the feasibility of recording with $\varphi 29$ DNAP as before, but with dissociation terms included in the forward kinetic model. Assuming a processivity of 70000 nucleotides—the value reported for $\varphi 29$ [55]—(processivity is defined as the inverse of the per-base probability of dissociation), we find that a re-association time (which scales with the polymerase concentration in solution) of >100 seconds still allows a median ion concentration estimation error of less than 1.5% under the same conditions found in Fig. 3A (N=1000, $E_0 = 0.005$ and $m = 0.025$). As a re-association time of >100 is much larger than realistic, we find that dissociation has a minimal effect. More generally, for a DNAP with similar kinetic parameters, even a low processivity of 100 nucleotides and a long re-association time of 10 seconds would allow 3.6% median ion concentration estimation error (Fig. S3). Therefore, dissociation is generally not a concern in these multi-condition experiments.

Sensitivity to start-time variation

We have also previously assumed that the polymerases are initially synchronized at the start of the recording experiment. Such perfect synchronization may be difficult to achieve in practice. We therefore examined the effect of asynchronous polymerase start-times. Using widely varying start-times (0-2 minutes; see *Text S4* for start-time distributions), we found a minimal effect on ion concentration estimation error (still <1.5%). Thus additional measures would not need to be taken to minimize DNAP start-time variation in multi-condition experiments.

Sensitivity to varying numbers of conditions

Lastly, we investigated how many conditions can be tested at high accuracy within an experimental timeframe of 20 minutes. This is equivalent to varying the temporal resolution at which firing rate estimation is attempted. Using $\varphi 29$ DNAP kinetic parameters (and N=1000, $E_0 = 0.005$ and $m = 0.025$), the median estimation error is 2.2% using 10 conditions, but jumps to over 7.1% using 15 conditions (Fig. S5A). However, DNAP parameters can be changed so that more conditions can be tested with small estimation errors. For example, when using 32 conditions, decoding with <5% median ion concentration estimation error is feasible for a DNAP with parameters $\tau_C \approx 5$ ms, $\tau_P \approx 800$ ms, $P \approx 0.01$ (Fig. S5B), which may be able to be constructed by combining favorable parameters from multiple natural polymerases. Additionally, increasing the total recording time could be used to increase the duration of each condition, increasing estimation accuracy. In general,

high accuracy estimation is feasible for up to approximately 10 experimental conditions when using a DNAP with kinetic parameters similar to $\varphi 29$ DNAP.

References

55. Blanco L, Bernad A, Lazaro JM, Martin G, Garmendia C, et al. (1989) Highly efficient DNA synthesis by the phage phi 29 DNA polymerase. Symmetrical mode of DNA replication. J Biol Chem 264: 8935-8940.
