# Supplementary material for: Statistical Analysis of Molecular Signal Recording
Source: PLoS Comput Biol. 2013 Jul 18;9(7):e1003145. doi: 10.1371/journal.pcbi.1003145 (PMC3715445; doi:10.1371/journal.pcbi.1003145)
Supplement: Text S3 — Importance of DNAP parameters. Discussion regarding how DNAP parameters affect the distribution of times at which nucleotides are written, and how this alters the resulting misincorporation probabilities. (PDF) [file pcbi.1003145.s009.pdf]

Text S3. Importance of DNAP parameters

Different parameters of nucleotide extension by DNAP can have a large effect on the shape of the time distribution at which nucleotides are written, even with the same mean replication rate. All three parameters (τ_C, τ_P, and *P*) affect the average speed with which DNA is replicated and thus the resulting misincorporation probabilities (Fig. S6A&D). Moreover, they each have different effects on the shape of the distribution, $\gamma_i(t;\theta_1)$. Longer regular extension durations (τ_C) primarily shift the distribution to the right. Longer pauses (τ_P) lead to a distribution with longer tails on the right hand side (Fig. S6B, green). The proportion of pauses (*P*) scales the weight of the tails (Fig. S6B, red). Thus, the specific, *in vivo*, dynamics of the polymerase can have profound implications on our ability to decode molecular ticker tapes. Fortunately, if a known signal can be applied, the molecular ticker tapes can be used to measure the behavior of the polymerase itself.
