# Supplementary material for: Statistical Analysis of Molecular Signal Recording
Source: PLoS Comput Biol. 2013 Jul 18;9(7):e1003145. doi: 10.1371/journal.pcbi.1003145 (PMC3715445; doi:10.1371/journal.pcbi.1003145)
Supplement: Text S4 — DNAP model. A general discussion about DNAP dwell time distributions and the assumptions made to produce our simplified model. (PDF) [file pcbi.1003145.s010.pdf]

Text S4. DNAP model

Correct or incorrect nucleotide addition by a DNAP requires multiple transitions between a series of intermediate conformational and chemical states, with associated forward and reverse kinetic rate constants that depend on the identity of the template nucleotide [56-58]. These processes give rise to several competing kinetic pathways, such as nucleotide addition, binding, pausing, dissociation, and exonucleolysis. In general, the dwell time of a polymerase can be modeled as:

$$\psi(t;\boldsymbol{\alpha},\boldsymbol{\phi})_{General} = \alpha_1\psi_1(t,\phi_1) + \ldots + \alpha_K\psi_K(t,\phi_K) \quad \text{(S4)}$$

for $t \geq 0$ [7], where each of the K alternative pathways has its own dwell-time distribution, $\psi_j(t,\phi_j)$ with kinetic parameters, ϕ_j, as well as a probability of occurrence α_j, where $\sum_{j=1}^{K}\alpha_j = 1$ for $\alpha_j \geq 0$. $\boldsymbol{\alpha} = \{\alpha_1 \ldots \alpha_K\}$ and $\boldsymbol{\phi} = \{\phi_1 \ldots \phi_K\}$. Some of the terms in *Eq. S4* can be ignored if the processes they represent are sufficiently rare and/or fast, as is done to make the two exponential model in the main text.

Initially, we ignore dissociation because the DNAPs used in molecular ticker tapes will ideally have very high processivity and/or be present at a sufficiently high concentration such that the kinetics of dissociation are considered negligible relative to those of association and re-association. Furthermore, it should also be possible to stop recordings after a short time and to impose a minimum-length selection on the synthesized strands, thus sequencing only template copies that were produced without any intermediate dissociation step. We also initially assume that the polymerases begin replication synchronously. We anticipate that *in vivo*

recording experiments will be synchronized through nucleotide starvation, small molecule modulators, or other engineered synchronization methods. Later in the main text, we explicitly analyze the effects of dissociation and start-time variation.

Many polymerases have 3'-5' exonuclease activity, which removes the base most recently added. This activity is ignored, as we assume that a polymerase lacking exonuclease activity will be used for recording molecular ticker tapes. Thus, our discussion applies only to polymerases with absent or greatly diminished exonuclease activity.

In addition, while DNAP pause times have been shown to follow an exponential distribution [6,51], to our knowledge, the distribution of elongation times has not been measured experimentally for DNAPs. Thus, our choice of an exponential distribution to represent elongation times is an approximation corresponding to the assumption that there is a single rate-limiting kinetic step in pause-free nucleotide addition. By the central limit theorem, for large numbers of nucleotides, the incorporation time distribution can be approximated as a Gaussian for most elongation time distributions, as we do in our simulations.

References

56. Joyce CM, Benkovic SJ (2004) DNA Polymerase Fidelity: Kinetics, Structure, and Checkpoints. Biochemistry 43: 14317-14324.

57. Xu C, Maxwell BA, Brown JA, Likui Z, Zucai S (2009) Global Conformational Dynamics of a Y-Family DNA Polymerase during Catalysis. PLoS Biol 7: e1000225.

58. Christian TD, Romano LJ, Rueda D (2009) Single-molecule measurements of synthesis by DNA polymerase with base-pair resolution. Proceedings of the National Academy of Sciences 106: 21109-21114.
