# Supplementary material for: Statistical Analysis of Molecular Signal Recording
Source: PLoS Comput Biol. 2013 Jul 18;9(7):e1003145. doi: 10.1371/journal.pcbi.1003145 (PMC3715445; doi:10.1371/journal.pcbi.1003145)
Supplement: Text S5 — Methods for testing simulations. Details are given regarding how all simulations are run. (PDF) [file pcbi.1003145.s011.pdf]

Text S5. Methods for testing simulations

In the simulations, for every DNA template copy, each nucleotide is chosen randomly to be a correct or incorrect addition, with the probability of being incorrect equal to that nucleotide's misincorporation probability, $P(\varepsilon_i;\boldsymbol{C},\boldsymbol{\theta_1},\boldsymbol{\theta_2})$ (see *Eq. 5*). Thus, N_i, the actual number of misincorporations at nucleotide *i* (see *Eqs. 8&9*), is simulated by counting how many of the simulated template copies have an incorrect replication of nucleotide *i*. Additionally, as discussed in the main text, in all simulations the concentrations are scaled to range from 0 to 1 (arbitrary units), so that *m* can be thought of as the maximum concentration-induced misincorporation probability change.

In our simulations, it's important to note when $\gamma_i(t;\theta_1)$, the distribution of incorporation times for nucleotide *i*, can be approximated as a Gaussian (*Eq. 4*). When using a DNAP without pausing, $\gamma_i(t;\boldsymbol{\theta_1})$ is approximately Gaussian for *i*>50. When using a DNAP with parameters near that of $\varphi 29$ DNAP ($\tau_C \approx 17$ ms, $\tau_P \approx 3000$ ms, $P \approx 0.025$), $\gamma_i(t;\theta_1)$ is approximately Gaussian for *i*>1000.

Continuous decoding

When determining the average ion concentration estimation error, randomly determined numbers between 0 and 1 are used for the concentration during each experimental condition. During testing, random concentration sequences are generated, copied templates are simulated, and the original sequence is decoded. The ion concentration estimation error during a condition *k* is:

$$Error = \left| \frac{\hat{C}_k - C_k}{C_k} \right|, \tag{S5}$$

where C_k are the randomly generated concentrations and $\hat{C}_k$ are the estimated concentrations.

Binary decoding

To test whether a set of experimental parameters were feasible at a particular temporal resolution (pulse duration) δ, and recording duration, τ_f, we determined whether the accuracy of decoding the concentration pulse, or "bit," from $\tau_f - \delta$ to τ_f was at least 95%. We estimated 10 bits, from $\tau_f - 5\delta$ to $\tau_f + 5\delta$, in order to realistically estimate the accuracy of decoding the bit from $\tau_f - \delta$ to τ_f (since decoding accuracy decays when estimating several bits). Each bit is randomly determined to be 0 or 1 (low or high concentration) with equal probability. During testing, random bit sequences are generated, copied templates are simulated, and then the original bit sequence is decoded.

DNAP dissociation from the template

When testing the effect of DNAP dissociation from the template on estimation of concentration traces, we add an additional exponential to our previous model (*Eq. 1b*) to represent a pathway in which the polymerase dissociates from the template to be later replaced by another DNAP:

$$\psi(t;\boldsymbol{\theta}_1,D,\tau_D)_{Dissociation}=P\cdot\frac{e^{-t/\tau_P}}{\tau_P}+D\cdot\frac{e^{-t/\tau_D}}{\tau_D}+\left(1-P-D\right)\cdot\frac{e^{-t/\tau_C}}{\tau_C}, \quad \text{(S6)}$$

where τ_D is the average time for the dissociating path (the time it takes the DNAP to re-associate) and *D* is the dissociation probability per nucleotide (inverse of processivity). When estimating $\gamma_i(t;\boldsymbol{\theta}_1)$ as a Gaussian distribution, we now have:

$$\mu_i = i\cdot\left(P\cdot\tau_P + D\cdot\tau_D + (1-P-D)\cdot\tau_C\right) \quad \text{(S7a)}$$

$$\sigma_i^{\,2} = i\cdot\left(2\cdot P\cdot\tau_P^{\,2}+2\cdot D\cdot\tau_D^{\,2}+2\cdot(1-P-D)\cdot\tau_C^{\,2}-(P\cdot\tau_P+D\cdot\tau_D+(1-P-D)\cdot\tau_C)^2\right) \quad \text{(S7b)}$$

With these distributions, the decoding accuracy can be tested as described above.

Start-time variation

We approximate the start-time variation as a Gamma distribution with parameters $(k,\theta)=(9,\mu/9)$, where μ is the mean of the distribution. This distribution was chosen because it is strictly positive, and roughly approximates a Gaussian with a standard deviation of $\mu/3$, so that almost all of the distribution is between 0 and 2μ. Thus, in the main text, when we say that we tested start-times that vary between 0 and 2 seconds, for example, we were representing the start-time distribution as a Gamma distribution with parameters $(k,\theta)=(9,\frac{1}{9})$. To find the time distribution of nucleotide incorporation that includes start variation, we convolve the original time distribution of nucleotide distribution with the time distribution of start-times. With this distribution, decoding accuracy can be tested as described above. For decoding, while the start-times are unknown for individual

templates, we assume that the distribution of start-times is known (this could be measured experimentally beforehand [17]).

Concentration fluctuations

To test the effects of fluctuating ion concentrations on ion concentration estimation, we allow concentrations to vary within an experimental condition, and determine the accuracy of estimating the mean concentration within that condition. Note that we are only concerned with estimating the mean concentration during a condition, not the dynamics of fluctuations, as our goal is to compare concentrations (corresponding to firing rates) between conditions. To compare estimation of "fixed" and "fluctuating" concentrations, we estimate fixed concentrations valued from 0.2 to 0.8 (estimated values can still be between 0 and 1). Fluctuating concentrations have a "baseline" concentration at each condition between 0.2 and 0.8, but the concentration value at every millisecond is chosen randomly from the interval [baseline-0.2 baseline+0.2].

Estimation with unknown DNAP parameters

In order to test the accuracy of estimating the DNAP dynamics parameters, we generate a predetermined time-varying concentration, and set the true parameter values. We then simulate N copied DNA sequences for a wide range of N's, and estimate the original parameters. We calculate the percent error of the estimated values relative to the true parameter values.

We test the validity of our algorithm by performing ion concentration estimation using the estimated polymerase parameters, rather than given parameters, as would be necessary when dealing with real experimental data. We first estimate the set parameter values. Next, we randomly generate a concentration trace, and simulate misincorporation counts based on that concentration trace and the true parameter values. Finally, we estimate this concentration trace using the estimated parameters in place of the true parameters. We evaluate how the previously determined number of template copies needed for perfect concentration decoding is affected by using estimated parameters rather than given parameters and determine how many template copies are needed in parameter estimation to allow the subsequent high-accuracy recovery of concentration traces.

Scalability

All simulation results in this paper are scalable on the time axis. When $\tau_C = w$, $\tau_P = x$, $T_0 = y$, and $\delta = z$, decoding results will be equivalent to when $\tau_C = \alpha w$, $\tau_P = \alpha x$, $T_0 = \alpha y$, and $\delta = \alpha z$.
